# Genomic assessment of targets implicated in *Rhipicephalus microplus* acaricide resistance

**DOI:** 10.1371/journal.pone.0312074

**Published:** 2024-12-05

**Authors:** Christina Meiring, Michel Labuschagne

**Affiliations:** 1 Clinglobal, Tamarin, Mauritius; 2 Clinomics, Bloemfontein, South Africa; 3 Department of Microbiology and Biochemistry, Faculty: Natural and Agricultural Sciences, Bloemfontein, South Africa; Beni Suef University Faculty of Veterinary Medicine, EGYPT

## Abstract

Globally, the prevalence of *Rhipicephalus microplus* resistance to various acaricides has increased, and there is a need for the identification of molecular markers that can predict phenotypic resistance. These markers could serve as alternatives to the larval packet test (LPT), enabling rapid and accurate monitoring of resistance in these ticks against multiple acaricides. However, many of the historically identified markers are present in isolates from specific countries and their role in acaricide resistance remains unclear. This study aimed to assess these mutations by sequencing genomic regions encoding proteins historically associated with acaricide target site insensitivity and increased acaricide detoxification and comparing resistant and susceptible isolates from eight different countries. Employing a novel multiplex PCR setup developed during the study, the coding regions of 11 acaricide-resistant targets were amplified and sequenced across 37 *R*. *microplus* isolates from different locations. The identified mutations, both previously reported and novel, were compared between acaricide-susceptible and acaricide-resistant isolates, phenotypically characterized using the larval packet test or larval immersion test across five acaricide classes. Genotypes were then correlated with available phenotypes, and protein modelling of novel nonsynonymous mutations was conducted to assess their potential impact on acaricide resistance. Previously reported resistance-associated mutations were detected, some of which were present in both resistant and susceptible isolates. Novel mutations emerged from the 11 targets, but distinctions between susceptible and resistant isolates were not evident, except for the prevalent *kdr* mutation in synthetic pyrethroid-resistant isolates. The quest for predictive molecular markers for monitoring acaricide resistance remains challenging. Nevertheless, by utilizing a representative group of isolates, we determined that several historical mutations were present in both resistant and susceptible isolates. Additionally, the study provides valuable genetic data on acaricide-resistant and susceptible isolates from different geographical regions, focusing on genomic regions implicated in resistance. This baseline data offers a critical foundation for further research and the identification of more reliable molecular markers.

## Introduction

*Rhipicephalus microplus* ticks cause significant economic losses by transmitting pathogens such as *Babesia bovis* among cattle. This results in decreased milk and meat production due to host anaemia or the loss of animals [1, 2]. Bovine babesiosis is considered the most economically important arthropod vector-borne disease of livestock in the world [3]. The control of tick infestations relies on the use of synthetic acaricides, which act mainly on the tick’s central nervous system through different mechanisms [4]. Most acaricides, such as synthetic pyrethroids (SPs), phenylpyrazoles, macrocyclic lactones, organophosphates (OPs), and formamidines, target various ion channels. These include the voltage-gated sodium channel, GABA-gated chloride channel, glutamate-gated chloride channel gene, acetylcholinesterase enzymes, and octopamine receptors [5], respectively (Table 1). The application of synthetic acaricides over the years has led to ticks becoming resistant to these compounds, and the high frequency of use and poor application practices can accelerate acaricide resistance selection in ticks [6, 7]. The observed acaricide resistance may be a result of mutations in the target binding site, increased degradation of the acaricide by detoxification pathways, and reduced uptake of the acaricide by the tick [5]. To assist with the minimization of resistance selection, there is a need for regular surveillance of ticks for resistance against acaricides [8].

**Table 1:** Two acaricide resistance mechanisms with the respective acaricide classes and their targets.

Mechanism of resistance	Acaricide Class	Acaricide Target
Target binding site resistance	Formadines	β-adrenergic-like octopamine receptor gene (βAOR)
		α-adrenergic-like octopamine receptor gene (αAOR)
		Octopamine-tyramine receptor gene (OCT/TYR)
	Macrocyclic lactones;	GABA-gated chloride channel gene (GABA-Cl)
	Phenylpyrazoles	Glutamate-gated chloride channel gene (Glu-Cl)
	Organophosphates	Acetylcholine esterase 2 (AChE2)
		Acetylcholine esterase 3 (AChE3)
	Synthetic Pyrethroids	Voltage-gated sodium channel gene (VGSC)
Metabolic resistance		Carboxylesterase gene (CBE)
		Cytochrome P450 CYP4W1
		Cytochrome P450 CYP41

Currently, there are bioassays available to evaluate tick resistance, which involve assessing tick mortality at different acaricide concentrations, such as the LPT and the larval immersion test (LIT) [9, 10]. Despite the many advantages of these bioassays, a major disadvantage is the time it takes to obtain a result (5–6 weeks) [11]. Additionally, these bioassays require live tick samples and are unable to detect genotypes of resistant individuals, thereby reducing the effectiveness of these techniques [8]. In recent years, many studies have focused on understanding the molecular basis of acaricide resistance, especially genetic mutations causing target-site modifications and increased metabolism of the acaricide [12, 13]. Identification of these genetic changes allows for the development of molecular assays, which have the advantages of providing results within a day and requiring fewer tick samples [8]. The development of molecular techniques for rapid resistance detection relies on the availability of accurate molecular markers that confer resistance to acaricides [14, 15].

Several mutations associated with acaricide resistance (of different acaricide classes) have been identified, and different types of molecular techniques have been developed to detect acaricide resistance in ticks [14, 16–18]. However, the exact role of these markers in conferring resistance is not always clear. Some may be involved in other biological processes or might be neutral variants, emphasizing the need for further investigation to identify more reliable and universally applicable resistance markers. Moreover, in many cases, molecular assays are expensive and need to be performed in a laboratory environment. Additionally, studies have shown that mutations detected in resistant *R*. *microplus* isolates are not solely responsible for the generation of phenotypic resistance, suggesting that additional mutations are likely to exist [19]. For some acaricide classes, multiple target genes are involved in resistance, adding to the complexity of developing effective diagnostic tools [9].

The understanding of the molecular mechanisms of resistance in ticks is still limited, but the availability of a high-quality *R*. *microplus* genome may provide more opportunities to overcome this [20]. Expanding the identification of the molecular targets involved in acaricide resistance is important for the development and improvement of molecular assays aimed at detecting acaricide resistance as early as possible [8]. This study employed a novel approach to sequence 11 target genes historically implicated in acaricide binding sites and detoxification resistance mechanisms against five acaricide classes in *R*. *microplus* larval tick isolates from eight different countries characterized by LPT or LIT. We expect that mutations (causing amino acid changes) that contribute to a resistance phenotype will be present at increased frequency in acaricide-resistant tick larval isolates relative to susceptible isolates.

## Results

### Sample pre-screening and selection

**Fig 1:**
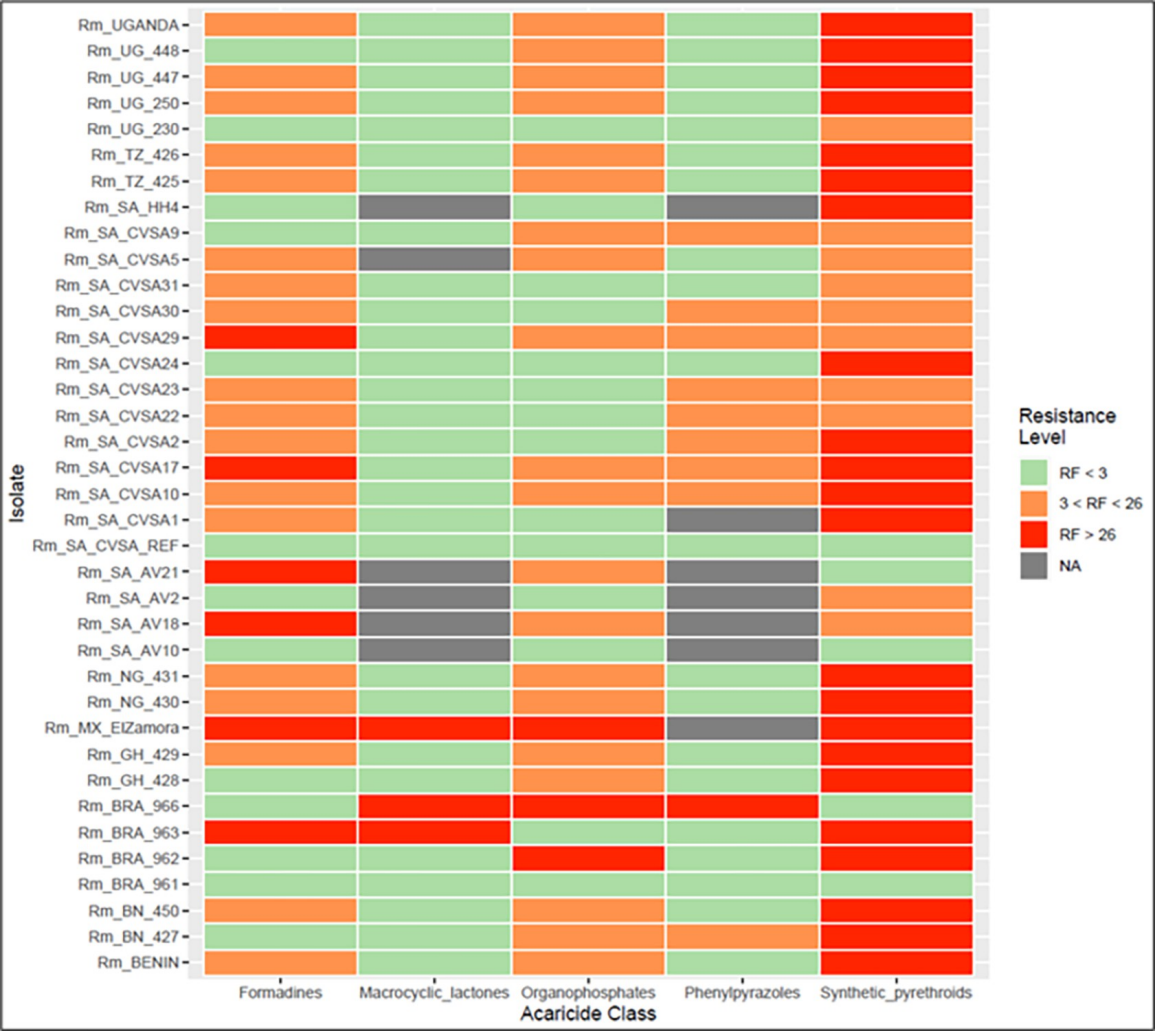
Heatmap indicating the resistance factors of 37 selected isolates for the five different acaricide classes [21]. The gray blocks (NA) indicate that no data are available for that sample.

The pre-screening analysis used to determine the species identity of tick isolates from different countries (*n* = 74) revealed that 48 isolates were pure *R*. *microplus*, whereas eight were identified as *R*. *decoloratus*. Additionally, six isolates exhibited a mixture of both species, whereas 12 isolates did not yield amplifiable DNA. On the basis of the acaricide resistance factors determined by the LPT test and the resistance or susceptibility classification from the LIT test (Fig 1), 37 out of the 48 confirmed *R*. *microplus* isolates were selected for target amplification and sequencing (S1 Table).

### Target identification and primer design

We successfully mapped and annotated coding sequences to the reference genome. This process enabled the identification of exon regions within the genomic target gene loci, ensuring accurate representation of the target sequences. The subsequent successful translation of these annotated regions provided strong validation of their alignment with the encoded protein sequences.

The evaluation of the designed oligonucleotides included the assessment of each primer pair separately against both the Clinvet South Africa *R*. *microplus* genome (RmCVSA) and geographically distinct *R*. *microplus* genomic DNA to confirm successful amplification. This approach, which resulted in a total of 13 multiplex primer mixtures, ensured the successful amplification of all 63 target amplicons, covering the entirety of the selected targets. (S2 Table).

### Target amplification and sequencing

The 11 genome targets were amplified from 37 *R*. *microplus* isolates using the designed multiplex PCR oligonucleotides (S2 Table), and the sequence reads for each isolate were mapped to the annotated targets. Consensus sequences were generated using a 75% threshold. If a position in the sequence was variable and no single nucleotide accounted for 75% or more of the reads, an ambiguous base (M, R, W, S, Y, or K) was used to indicate uncertainty. Positions with high variability where no clear consensus could be determined were assigned an ‘N,’ indicating an unknown or undetermined nucleotide. This ambiguity mostly resulted in an unknown (X) amino acid at those positions during translation.

Introns within the consensus sequences were removed, and the exons for all the isolates were multiple aligned per target. The multiple sequence alignments were translated and compared between the acaricide-susceptible and acaricide-resistant isolates to identify amino acid substitutions in each target (S3 Table).

#### Octopamine receptor genes (αAOR; βAOR; OCT/TYR)

Using our mapping approach, we successfully identified all three classes of octopamine receptors in the RmCVSA genome. We obtained full-length coding sequence data for each target and were able to amplify the exons from all the isolates included in the study via PCR.

Previously reported nonsynonymous mutations in βAOR in relation to amitraz resistance were not detected in isolates from this study. Two novel non-synonymous substitutions were identified in the coding region of the β-adrenergic-like octopamine receptor gene (βAOR) that have not been reported in other *R*. *microplus* isolates (S3 Table; S1A Fig). One substitution, A104V, was observed in a single sequence (Rm_MX_ElZamora) and is known to be an amitraz-resistant isolate [22, 23]. Additionally, five isolates had an amino acid substitution at position C319G (S1A Fig), all of which had amitraz resistance factors above 3. The A104V and C319G mutations were mapped to the second and the sixth transmembrane domains, respectively (S1B Fig). Previous investigations comparing the amitraz/DPMF sensitivity of *Varroa destructor* mite and honeybee βAOR sequences indicated that three residues (E208; I335; I350) near the predicted amitraz/DPMF binding site in the honeybee βAOR contributed to the observed resistance [24]. Multiple sequence alignment of the *R*. *microplus* and *V*. *destructor* βAOR proteins allowed identification of the *R*. *microplus* βAOR putative amino acid homologues involved in substrate binding. Notably, the C319G mutation is directly adjacent to W320, which is part of the putative substrate binding pocket (S2 Fig).

Currently, there are no data available in the literature on the α-adrenergic-like octopamine receptor gene (αAOR) target in relation to amitraz resistance. Multiple sequence alignment of the *R*. *microplus* αAOR gene product revealed four novel amino acid changes (S3A Fig), none of which were exclusively observed in amitraz-resistant isolates (RF > 3). Mapping these substitutions onto an AlphaFold 3D model of αAOR revealed that they are located remotely from the putative target binding site and reside in flexible loop regions of the intra- and extracellular domains (S3B Fig).

Protein sequence alignments identified previously reported amino acid substitutions (T8P, I15V, T20A, and L22S) in the Octopamine–tyramine receptor gene (OCT/TYR) gene across several isolates (S4 Fig) [5, 25, 26]. All four substitutions were present in a single amitraz-resistant isolate, Rm_SA_CVSA17 (RF > 26), which also had an additional M26T substitution. This M26T substitution was identified in 9 other isolates with amitraz RFs ranging between 0.50 and 89.81. The Rm_MX_ElZamora (RF > 26) isolate also had I15V, T20A, and M26T substitutions plus three novel substitutions (G294D, E311Q, and N414H). The T20A substitution was present as a heterozygous SNP in most isolates and was spread across the resistance factor range. All three isolates with the I15V substitution also had the T20A and M26T substitutions.

Mapping of historical and newly discovered mutations in the *R*. *microplus* OCT/TYR receptor to the AlphaFold 3D structure revealed that all historical mutations and the M26T mutation were located in the extracellular N-terminal region. In contrast, G294D, E311Q, and N414H were positioned on the intracellular C-terminus. (S5 Fig).

#### GABA and glutamate-gated chloride channel genes (GABA-CL; Glu-Cl)

Historical nonsynonymous mutations linked with fipronil resistance were not detected in our isolates. A single novel mutation was detected in the GABA-gated chloride channel gene (GABA-Cl) channel gene across 30 *R*. *microplus* isolates (not all the isolates had fipronil RFs available) (Fig 1; S6A Fig). This heterozygous mutation (G52R) resulted in 13 out of 30 isolates having the isoleucine/valine (I/V) amino acid at position 18. Given that the majority of isolates were susceptible to macrocyclic lactones, we did not explore the associations between this substitution and the RFs of ivermectin and doramectin. Mapping the known mutation to the predicted 3D monomeric structure of GABA-Cl (S6B Fig) revealed that the V18I mutation was present in an N-terminal α-helix. Further analysis indicated that the first 23 residues are predicted to be part of a signal peptide (predicted by SignalP 6.0), which will be removed, resulting in the mature monomer assembling into the mature pentameric GABA-Cl channel without the signal peptide region.

DNA sequencing of the glutamate-gated chloride channel gene (Glu-Cl) channel gene revealed several mutations present at the nucleotide level, but none resulted in any amino acid substitution in any of the isolates evaluated in this study. Additionally, we did not find any resistance-associated substitutions related to the Glu–Cl channel in the literature.

#### Acetylcholine esterase genes (AChE2; AChE3)

Four previously reported amino acid substitutions (V297I, S364T, H412Y, and R468K) in the acetylcholine esterase 2 (AChE2) gene were observed in all 37 isolates from this study (S3 Table; S7 Fig), including those classified as susceptible based on a chlorfenvinphos RF of ≤ 3 [5, 8].

Several additional amino acid changes were observed, with non-synonymous changes between residue positions 12 and 195 (S7 Fig) primarily appearing in the same 10 isolates, all of which presented resistance factors above 3 to chlorfenvinphos (RF > 3). Notably, the V12A, K22E, A50S, E57D, T/A290A, N/D296D, and R303T substitutions (S7 Fig; red font) were present simultaneously in three isolates (Rm_UGANDA, Rm_NG_430, and Rm_MX_ElZamora). Other isolates exhibited heterozygous genotypes at these positions. Additionally, six novel non-synonymous substitutions were identified in three isolates (Rm_BENIN, Rm_TZ_426, and Rm_MX_ElZamora), including T269N, N355Y, E453D, E489D, G492S, and R502K (S7 Fig, blue font; S8 Fig, blue residues). These isolates also had the T290A and R303T substitutions.

Several heterozygous mutations were detected in this gene, primarily in isolates with resistance factors less than 3 chlorfenvinphos. The isolates with the most shared mutations were Rm_BENIN and Rm_UG_447 (16 mutations), followed by Rm_UG_448, Rm_GN_429, and Rm_TZ_426 (14 shared mutations each), and Rm_MX_ElZamora (13 concurrent mutations). All these isolates had OP resistance factors ≥ 7.7.

Mapping these highlighted mutations (S7 Fig; blue font) to the AlphaFold-predicted 3D structure (S8 Fig) indicated that all but one of the mutations (blue) are located in flexible regions on the surface of the structure. The N355Y mutation is situated directly next to E356, the acidic member of the catalytic triad (S230—E356—H476).

In the acetylcholine esterase 3 (AChE3) gene, five non-synonymous substitutions (relative to the reference sequence) were identified, all of which have been reported in *R*. *microplus* isolates from other studies (I54V, R86Q, V137I, I492M, and T548A) (S3 Table; S9 Fig) [8, 27–29]. Two of these amino acid changes (R86Q and I492M) were present in all 37 isolates from this study. The I54V substitution was detected in all but one isolate, V137I was detected in 23 isolates, and T548A was detected in 23 isolates.

Mapping these mutations on a 3D model of AChE3 (S10 Fig) revealed that all the mutations except I492M were located on the surface of the enzyme structure. The I492M mutation is located in the inner core of the protein, close to the active site, with a distance of 4 to 7 Å between the side chains of I492M and the catalytic triad members.

#### Voltage-gated sodium channel gene (VGSC)

**Fig 2:**
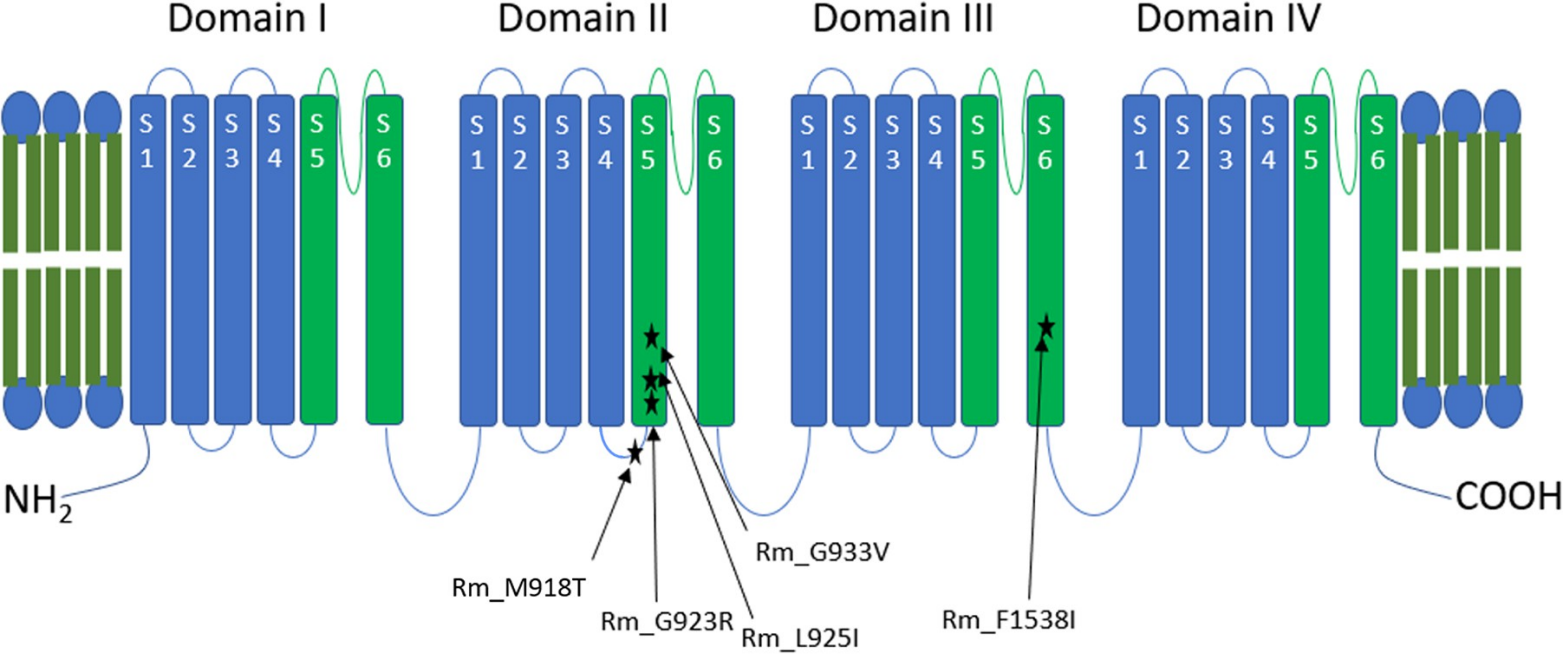
Schematic representation of the *R*. *microplus* VGSC highlighting pyrethroid resistance conferring target-site mutations. The topology of the VGSC shows amino acid substitutions and their positions relative to the five transmembrane domains, each containing six membrane-spanning segments. The membrane-spanning segments are colour-coded: blue for the voltage-sensing modules and green for the pore modules. All residue numbers correspond to positions in the house fly VGSC (GenBank accession: X96668).

The voltage-gated sodium channel (VGSC) in *R*. *microplus* was recently reviewed and found to contain five amino acid substitution sites associated with pyrethroid resistance, located in domains II and III (Fig 2) [5, 20].

**Fig 3:**
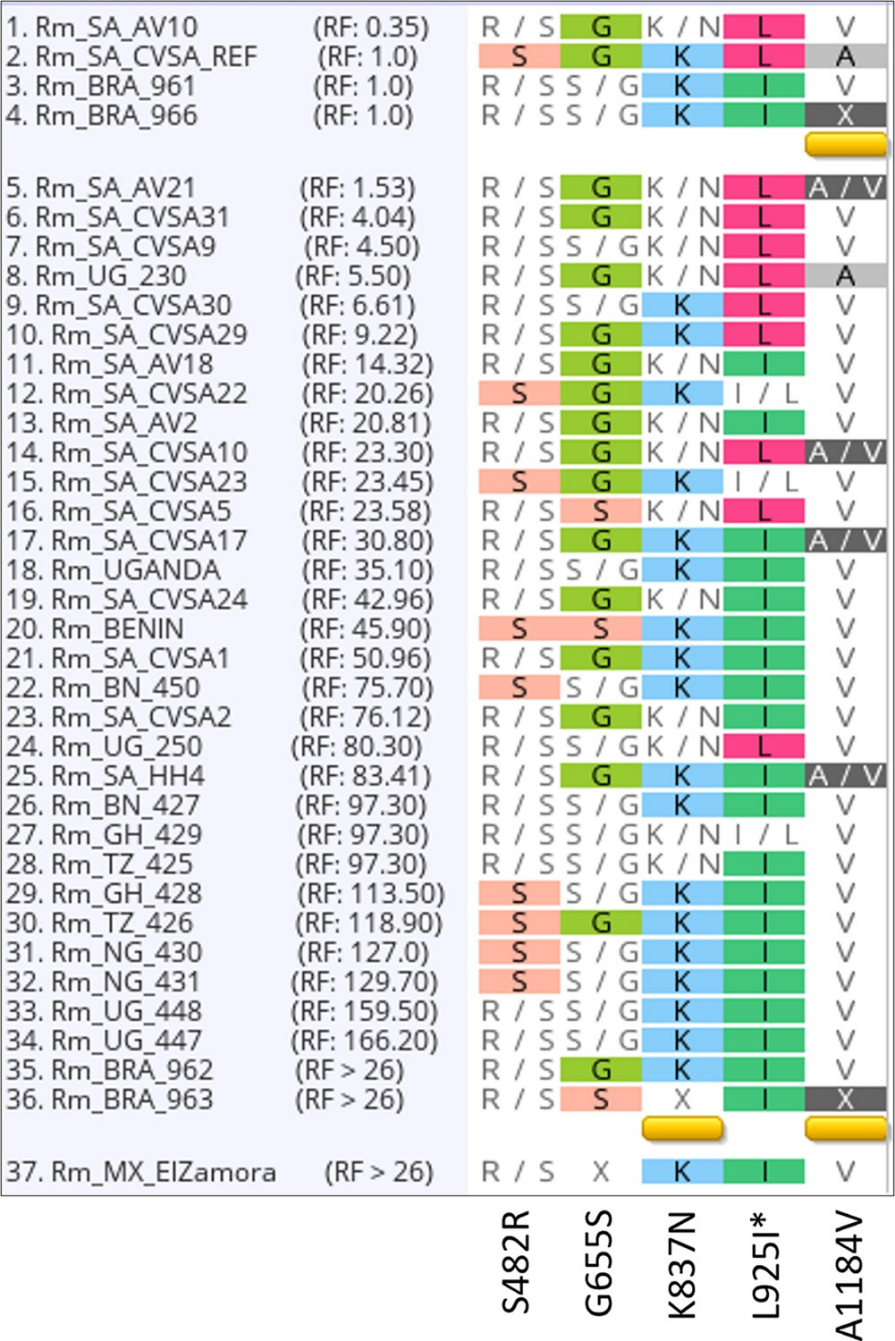
Multiple sequence alignment of amino acid substitutions in the VGSC gene in *R*. *microplus* isolates. The isolates are ordered by their synthetic pyrethroid resistance factor (RF) (deltamethrin; alpha-cypermethrin), with isolates having the lowest RF at the top and the highest RF at the bottom. Isolates Rm_BRA_962, Rm_BRA_963, and Rm_MX_ElZamora, which did not have exact RF values, are labelled RF > 26, as they were confirmed to be phenotypically resistant to SP. The ‘X’ at position 655 in the Rm_MX_ElZamora isolate indicates high variability, leading to more than two possible amino acids at that position. All other ’X’ amino acids indicate a lack of sequence data at those positions. *Denotes previously reported mutations.

The mutation initially associated with *kdr*, causing the amino acid change from leucine to isoleucine (L925I) in *R*. *microplus*, was observed in 23 isolates from this study (Fig 3). An additional three isolates had a heterozygous genotype at this position. Most of these isolates (20 isolates) had SP resistance factors above 26 (RF > 26). Furthermore, 28 isolates had heterozygous mutations resulting in an amino acid change from serine to serine/arginine at position 482. Additionally, 29 isolates had a novel substitution at position 1184, leading to a change from alanine to valine (A1184V), with four other isolates being heterozygous for this mutation (S3 Table; Fig 3). The S482R and A1184V amino acid substitutions did not occur more frequently in the SP-resistant isolates than in the SP-susceptible isolates, suggesting that they may not contribute to phenotypic resistance to this acaricide. This might also be true for the heterozygous mutation observed in 12 isolates, which led to a lysine/asparagine amino acid change at position 837 (Fig 3). Finally, 16 isolates presented a serine/glycine amino acid at position 655, and 3 isolates presented a substitution causing the amino acid to change from serine to glycine (G655S), which has not been reported in previous studies.

**Fig 4:**
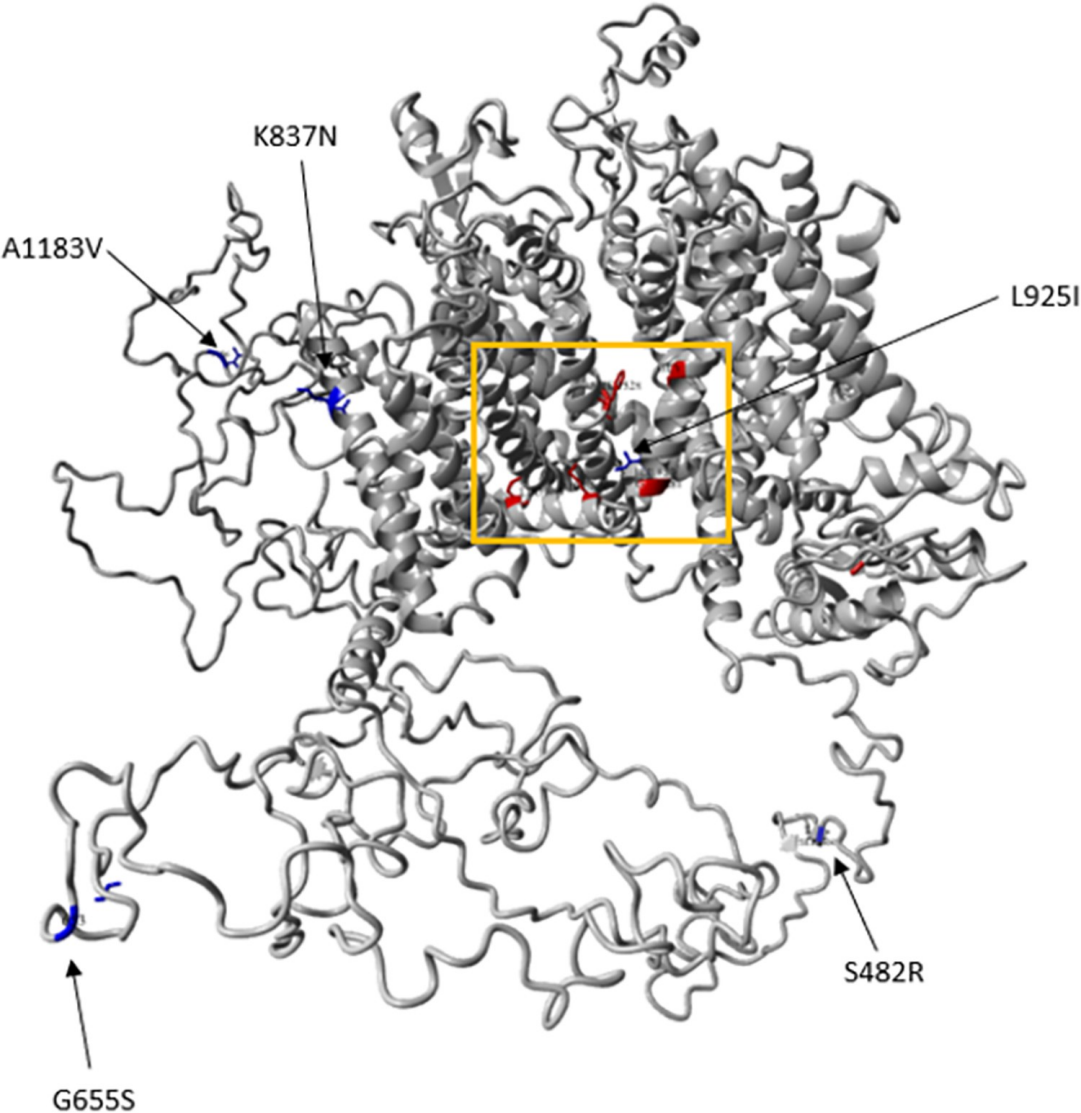
3D structure of *R*. *microplus* VGSC with key mutations and deltamethrin binding. SwissModel 3D structure of the *R*. *microplus* VGSC protein with amino acid substitution sites (as listed in Fig 3), mapped in blue. The PyR1 binding site and the amino acid substitutions known to be associated with *R*. *microplus* pyrethroid resistance (Fig 2; mapped in red in Fig 4) are located inside the orange rectangle. All residue numbers correspond to the position of the house fly sodium channel (GenBank accession: X96668).

Mapping the amino acid substitutions identified in this study onto the 3D model of the *R*. *microplus* VGSC protein demonstrated that only L925I is located at the pyrethroid binding receptor 1 (PyR1) site (Fig 4; orange rectangle). This highly conserved L925I substitution has been functionally confirmed in *Xenopus* oocytes, resulting in decreased sensitivity to deltamethrin, permethrin, fenfluthrin, and DDT [30]. The other substitutions (S482R; G655S; A1184V) are located in loop regions, with K837N present in voltage-sensing domain II. This mutation has not been reported in the literature, mainly since most studies have focused on the historical identification of mutations in the domain II S5 region, which is easier to amplify and sequence via Sanger-based workflows.

None of the other substitutions found in this study could be assigned to any of the regions implicated in insect VGSC modelling to assess the impact of PyR1, PyR2, and regions beyond these binding sites, as determined by an extensive analysis of the locations of mutations on the insect VGSC and their impact on pyrethroid binding [31].

#### Carboxylesterase gene (CBE)

We identified a previously documented carboxylesterase point mutation resulting in an amino acid change from aspartic acid to asparagine (D374N). This mutation was detected in 14 isolates, predominantly in a heterozygous state (S3 Table; S11 Fig), and was observed more frequently in isolates with a high level of SP resistance. Additionally, nine concurrent amino acid substitutions were present in 15 isolates with SP RFs ranging between 35.10 and 166.20 (S11 Fig; blue font) as well as in two isolates from Brazil with susceptible phenotypes.

Furthermore, since the D374N marker has also been associated with OP resistance in *R*. *microplus* ticks [32, 33], we examined its frequency in OP-resistant isolates compared with OP-susceptible isolates. We found that it was present primarily in resistant isolates (S12 Fig). The amino acid profiles of the SP-resistant and susceptible isolates (S11 Fig) were similar to those observed for the OP resistance factors (S12 Fig).

Mapping the identified CBE substitutions to the predicted 3D structure indicated that most substitutions are located on the outer surface of the protein (S13A Fig). Docking deltamethrin to the *R*. *microplus* CBE (S13B Fig) revealed that the Q/H465Q substitution was located close to the active site and within 2.5 Å of the docked substrate.

#### Cytochrome P450 genes (CYP4W1; CYP41)

Mutations in cytochrome P450 CYP4W1 and CYP41 have been reported in *R*. *microplus* ticks, some of which were identified in isolates from this study. Three (A74T, I539K, and V544E) of the five amino acid substitutions detected by Nagar et al. [34] in an SP-resistant isolate were present in all 37 isolates evaluated in this study (S14A Fig). When mapped to the predicted 3D structure, all three mutations were located on the outer surface of the protein, with I539K and V544E located close to the surface-exposed C-terminal end (S14B Fig).

A novel heterozygous mutation was identified in 4 isolates, including two SP-susceptible isolates, resulting in an E396D substitution. This substitution was mapped to a surface loop region of the protein. Given the location and similar characteristics of the replaced residue, it is not anticipated to contribute significantly to acaricide resistance.

Since cytochrome P450 genes are associated with OP resistance in *R*. *microplus* ticks, we examined amino acid changes in OP-resistant isolates compared with OP-susceptible isolates (S15 Fig). A similar trend was observed, with substitutions present in susceptible and resistant isolates.

Six (T224A, T244S, N248D, G388A, T389S, and C484R) of the ten previously documented amino acid substitutions in the CYP41 gene were also identified in isolates from this study (S16 Fig) [34]. The Rm_BENIN isolate had heterozygous mutations resulting in a threonine/serine (T/S) amino acid at position 244 and an asparagine/aspartic acid (N/D) amino acid at position 248. This amino acid change occurred at position 248 in nine other isolates. The T224A, G388A, T389S, and C484R substitutions were detected in all 37 isolates included in this study (S16 Fig). The Rm_BRA_966 isolate (SP-susceptible) had novel mutations causing amino acid changes at positions 155, 289, and 304. Additionally, 35 isolates had a glutamic acid/aspartic acid (E/D) substitution at position 266.

We also examined amino acid alterations in OP-resistant isolates compared with OP-susceptible isolates and reported that substitutions were present across both susceptible and resistant isolates (S17 Fig).

Mapping the amino acid substitutions detected in this study onto the predicted 3D structure of *R*. *microplus* CYP41 indicated that most of the substitutions were located on the outer surface of the protein, distant from the heme and substrate binding sites (S18 Fig). The G388A and T389S substitutions, which result in amino acid changes with similar characteristics and are present in all the isolates tested, are located directly adjacent to R390 and E392, which are implicated in substrate binding, and L391 and R393, which are involved in heme binding [35].

## Discussion

In this study, we aimed to sequence the coding regions of 11 protein targets historically associated with acaricide resistance. We developed a multiplex PCR strategy capable of simultaneously amplifying all designated targets and used a DNA-based amplicon sequencing approach to investigate the presence of non-synonymous mutations. The resulting data were used to explore the prevalence of previously documented mutations in our *R*. *microplus* isolates and to compare novel mutation frequencies between resistant and susceptible isolates. Genomic loci containing the encoding genes were identified by mapping the coding sequences to the RmCVSA genome. The primers were designed to amplify the exons with a minimal number of primer pairs while accommodating partially degraded DNA. The 63 primer pairs were used in 13 multiplex PCRs to amplify all the exons of the 11 target genes.

This multiplex PCR setup was used to amplify targets from 37 geographically diverse *R*. *microplus* isolates with LPT/LIT phenotypic data. Sequence analysis revealed the complete coding sequences of the targets, providing a collection of complete coding sequences of geographically diverse *R*. *microplus* isolates with resistance phenotypes. This DNA-based workflow offers an alternative to RNA-based target sequencing, allowing for the detection of all targets without requiring tissue-specific samples for amplicon generation and sequencing. However, this approach does not account for the differential expression of detoxification enzymes.

The analyses provided deeper insights into historical mutations reported in the literature and revealed numerous novel substitutions within the coding sequences of the acaricide resistance associated target regions. Having multiple susceptible control isolates from diverse geographic locations for all the different acaricide classes enabled us to account for genetic variation within the different isolates and eliminate the variants, if present, as possible resistance-causing mutations.

### Octopamine receptor genes (αAOR; βAOR; OCT/TYR)

Octopamine is a crucial neuromodulator that regulates cellular cAMP and calcium levels through its interaction with octopamine receptors [36]. Amitraz, a broad-spectrum formamidine acaricide, targets these receptors, activating pathways that control the cAMP and calcium levels in the cell [37]. These receptors are part of the G protein-coupled receptor family and are characterized by seven transmembrane segments, an intracellular C-terminal domain, and an extracellular N-terminal domain. They can be categorized into αAOR, βAOR, and OCT/TYR [36, 38]. Although the specific molecular targets of amitraz and its active metabolite DPMF in Acari are not completely understood, recent studies suggest that βAOR is the primary target [20, 24].

Recently, the I61F mutation in the first transmembrane domain of *R*. *microplus* βAOR was identified and associated with amitraz resistance [39]. This mutation reduces sensitivity to DPMF when introduced into the orthologous *Bombyx mori* βAOR [40]. In this study, none of the isolates showed genetic variation at the I61F codon, indicating that high resistance factors (RF > 10) must be associated with mutations other than I61F. Additionally, no consistent association with the C319G mutation in the sixth transmembrane segment was found in isolates exhibiting an RF > 10 for amitraz (S1 Fig). However, the proximity of C319G to the W320 residue, a critical component of the predicted substrate-binding pocket, suggests that C319G may play a significant role in modulating the interaction of the receptor with amitraz, potentially contributing to resistance mechanisms in *R*. *microplus*.

The substitutions identified in this study, which were mapped onto an AlphaFold 3D model of αAOR, were located far from the putative target binding site. The S28C substitution, situated within the first transmembrane segment, is in a region lacking any substrate-binding pocket residues, making it unlikely to contribute significantly to amitraz resistance. The literature also indicates that while αAOR can be activated by amitraz/DPMF, only βAOR leads to measurable in vivo effects due to the presence of the agonist [24].

In the OCT/TYR gene, we identified eight amino acid substitutions predominantly present in amitraz-resistant isolates, warranting further investigation into their potential role in phenotypic resistance to amitraz. However, the current phenotypic/genotype correlation between previously reported amino acid substitutions (T8P, I15V, T20A, and L22S) and amitraz resistance remains unknown [20].

A novel non-synonymous mutation identified in this study, leading to the M26T substitution, was present at an increased frequency in amitraz-resistant isolates relative to susceptible isolates, suggesting its potential role in resistance. The G294D, E311Q, and N414H substitutions, although present in a single known amitraz-resistant isolate, should be monitored in future investigations.

Mapping these substitutions to the AlphaFold 3D structure of the OCT/TYR receptor revealed their location within flexible loop regions of the peptides, which were distant from the predicted ligand-binding site within the transmembrane cavity. This makes a direct contribution to the observed amitraz resistance highly unlikely.

### GABA and glutamate-gated chloride channel genes (GABA-Cl; Glu-Cl)

GABA-Cl and Glu-Cl channel receptors are targeted by phenylpyrazoles (fipronil) and macrocyclic lactones, which inhibits the movement of chloride ions through these channels [5, 41–44]. Resistance to fipronil in *R*. *microplus*, linked to GABA-Cl receptors, emerged within 10 years of its initial use in Uruguay [45]. The A286S/L substitution (homologous to dieldrin resistance) is associated with fipronil resistance, with additional substitutions detected in resistant isolates containing the A286S substitution: S281T, A286L/S, V317I, T328A, and A329S [46]. In this study, we identified only one substitution in the GABA-Cl channel gene (V18I). This substitution was distributed across a wide range of RFs, making establishing any clear association with the observed phenotypes challenging.

Several mutations were detected within the Glu-Cl exons, but none resulted in any amino acid substitution in any of the isolates evaluated in this study.

### Acetylcholine esterase genes (AChE2; AChE3)

Organophosphates bind and inhibit acetylcholine esterase enzymes, preventing the degradation of the neurotransmitter acetylcholine. This inhibition results in continuous neuron firing, ultimately leading to death [20]. Observations have revealed an accumulation of mutations in more OP-resistant isolates, including the known OP-resistant isolate Rm_MX_Elzamora [22, 23, 47]. This accumulation suggests that these mutations, when combined, may play a role in the development of the OP-resistant phenotype. Previous studies on insects have investigated the interaction of multiple mutations in AChE genes and reported that these mutations have an additive effect on resistance [4, 48, 49].

Concurrent amino acid substitutions were observed in the isolates, and mapping these substitutions onto the AlphaFold-predicted 3D structure (S8 Fig) revealed that the N355Y mutation is located adjacent to E356, an acidic residue in the catalytic triad (S230—E356—H476). This exchange of an uncharged polar side chain with one containing an aromatic ring could be of interest because of its proximity to the active site and the potential for aromatic ring interactions between the tyrosine ring and the aromatic ring structure of chlorfenvinphos. Further insight could be gained by performing in-depth docking and molecular dynamic simulations of the chlorfenvinphos ligand within the active sites of the wild-type (WT) and mutated (MUT) enzymes.

The four previously reported substitutions in AChE2 were observed in five OP-resistant *R*. *microplus* isolates from India and were absent in a susceptible isolate [27]. This observation was based on diazinon resistance rather than chlorfenvinphos resistance. However, the presence of these mutations in all the isolates from various geographical locations assessed in this study suggests that these mutations are not associated with higher survival rates in response to chlorfenvinphos and may not contribute to the organophosphate (OP)-resistant phenotype. These findings indicate that different mechanisms of resistance may involve various OP compounds, highlighting the complexity of resistance and the need for further investigations into other genetic or biochemical factors contributing to OP resistance.

In the AChE3-encoding gene, the five identified amino acid substitutions occurred in both the resistant and susceptible isolates, including the chlorfenvinphos reference isolates (Rm_SA_CVSA_REF, Rm_BRA_961, and Rm_BRA_963). While these substitutions have been reported in other *R*. *microplus* isolates, their consistent presence across our isolates suggests that they are unlikely to be associated with resistance and are likely from a different genetic lineage. For example, the R86Q mutation has demonstrated the ability to generate an OP-resistant enzyme in vitro [29], and its presence in both resistant and susceptible isolates indicates that this mutation alone is insufficient to confer an organophosphate-resistant phenotype. Several amino acid substitutions have been identified within this target across isolates from various countries, yet none have been found exclusively in resistant isolates. These findings suggest that the development of the organophosphate-resistant phenotype likely involves additional mutations within the AChE3 gene, other AChE genes, or alternative mechanisms such as metabolic resistance [29].

### Voltage-gated sodium channel gene (VGSC)

The VGSC serves as the binding target for pyrethroids, inducing rapid paralysis in target species by causing prolonged channel activation and blocking action potential conduction. Mutations in the VGSC result in "knockdown" resistance, commonly known as *kdr*. Over 50 VGSC mutations have been identified in target species exhibiting resistance to pyrethroids [50]. Among the five amino acid substitutions identified in the isolates from this study, only the L925I substitution (*kdr*) was more prevalent in resistant isolates than in susceptible isolates. However, inconsistencies were observed, particularly in cases where isolates exhibited a high RF without the *kdr* substitution, such as Rm_UG_250. These findings suggest the potential involvement of an alternative mechanism in conferring phenotypic resistance to synthetic pyrethroids. The presence of the *kdr* substitution L925I among diverse isolates from different continents aligns with the literature [5, 20], confirming its widespread occurrence.

Interestingly, neither the *super-kdr* (super-knockdown resistance) mutation (M918T) nor the F1538I mutation (Fig 2) was identified in any of the isolates from this study, even those with extremely high resistance factors to deltamethrin and alpha-cypermethrin. These mutations have been identified in *R*. *microplus* isolates from the USA, Mexico, Brazil, and India (M918T only) but have not been reported in South Africa or other African countries to date [20, 51–53]. Furthermore, the G933V substitution was also absent in our isolates. This mutation has been identified in *R*. *microplus* isolates from Australia, Sri Lanka, Brazil, and Mexico [51] and is specifically linked to flumethrin resistance rather than cypermethrin resistance [54]. Finally, the G923R substitution, which has been identified in pyrethroid-resistant isolates from the USA and Mexico, was also not detected in any of the isolates from this study [51, 52].

### Carboxylesterase gene (CBE)

The *R*. *microplus* CBE protein is a key component of the metabolic acaricide resistance pathway. This pathway can contribute to resistance through the upregulation of mRNA transcripts encoding metabolic proteins and/or through point mutations in the protein, which increase its activity and specificity towards the targeted acaricide [4, 8]. Previous studies on the role of carboxylesterase in SP resistance have revealed that mutations within this gene may increase esterase hydrolytic activity towards SPs [55]. Specifically, a point mutation in this gene (G1120A), resulting in an amino acid change from aspartic acid to asparagine (D374N), has been reported more frequently in strains resistant to SPs than in susceptible isolates, suggesting its role in the development of acaricide resistance [8, 32, 56].

Interestingly, this mutation was absent in South African isolates, despite some of these isolates having high RFs (> 80) towards deltamethrin. Compared with isolates from other regions, South African isolates displayed a distinct amino acid substitution profile (S11 Fig), with the A334T amino acid substitution (threonine/alanine) being the only common change found in both South African and other international isolates. The novel T/A378T substitution, predominantly present in non-South African isolates, was located just four residues away from the historically significant D374N substitution on the same α-helix, as mapped onto the predicted 3D structure of *R*. *microplus* CBE. This T/A378T substitution warrants further investigation because of its close proximity to the D374N substitution. Additionally, the Q/H465Q substitution could also be of interest because of its close proximity to the active site. The Q to H substitution introduces an imidazole ring structure, allowing for π‒π stacking interactions between the imidazole ring and the phenyl ring in the substrate, as well as interactions with several nearby residues through ring interactions [57].

The distinct CBE substitution patterns observed in the SP-resistant isolates from South Africa are likely due to the introduction of *R*. *microplus* isolates with distinct genetic backgrounds into the country over time. The substitution patterns in the South African isolates revealed a hybrid background, combining different patterns present in all the isolates evaluated in this study.

### Cytochrome P450 genes (CYP4W1; CYP41)

Cytochrome P450 monooxygenases have been shown to be involved in metabolic resistance through their ability to detoxify pyrethroids [34, 58]. As integral components of metabolic acaricide resistance pathways, these enzymes can contribute to resistance through two primary mechanisms: the upregulation of enzyme production (transcriptional upregulation) and the accumulation of advantageous mutations. These mutations can increase the enzyme’s biological activity and increase its efficacy in the metabolism of specific acaricides.

The presence of the three previously reported substitutions in all 37 isolates evaluated in this study suggests that these mutations led to non-deleterious substitutions. These substitutions do not indicate enhanced fitness towards the SP or OP. A similar trend was observed for the substitutions identified in the CYP41 gene, where all the observed changes were found in the SP-susceptible isolates. Four previously identified changes, namely, T224A, G388A, T389S, and C484R, were consistently present across all 37 isolates and are not presumed to contribute to the observed resistance.

## Conclusion

This study presents a comprehensive workflow that enables researchers to amplify multiple acaricide resistance targets within their own laboratory settings using the provided primer sequences. These primers demonstrated their efficacy across a diverse range of isolates, successfully amplifying all designated targets from various geographic sources. They also allow for single-target amplification, facilitating efficient screening of many isolates through standardized amplicon barcoding. This approach overcomes the logistical challenges associated with importing and exporting tick isolates by utilizing readily accessible resources and consumables, thus eliminating the need to send samples elsewhere for processing. The only prerequisite for this workflow is the acquisition of Oxford Nanopore sequencing technology, with the MinION available at a relatively low cost.

The complete workflow offers a comprehensive sequence-based alternative to traditional methodologies and can be applied universally to any DNA target from any organism for amplicon-based sequence analysis. Importantly, this study revealed that previously reported mutations were present in both resistant and susceptible isolates, indicating that these mutations should no longer be considered in isolation as predictive markers for acaricide resistance in *R*. *microplus* ticks. Consequently, future research should focus on identifying other genetic factors contributing to resistance and perhaps go beyond investigating target-site resistance. This workflow provides reference target data for *R*. *microplus* ticks, aiding in understanding the molecular mechanisms of resistance in ticks.

## Materials and methods

### Sample pre-screening and selection

Tick isolates from South Africa, Brazil, Tanzania, Benin, Ghana, Nigeria, Uganda, and Mexico were obtained (*n* = 74) and subjected to a pre-screening analysis to confirm whether the isolates are of the *R*. *microplus* tick species. DNA was extracted from a pool of 25 larvae from each isolate, followed by PCR amplification of the internal transcribed spacer 2 (ITS2) region using the following primers: Tick_uni_ITS2-1F, 5’-GTCGGCAACACGGACAGCACGCTGAAC-3’ and Tick_uni_ITS2-1R, 5’-GACCGACGGCGGACTACGACG-3’. The PCR conditions were as follows: initial denaturation at 98°C for 2 minutes, followed by 30 cycles of 98°C for 10 seconds, 60°C for 20 seconds, and 72°C for 2 minutes, with a final extension at 72°C for 5 minutes. The PCR amplicons were sequenced using the Oxford Nanopore Technology (ONT) sequencing platform and subjected to GPU guppy base calling. The sequence reads for each isolate were mapped to *R*. *microplus* as well as the *R*. *decoloratus* ITS2 sequence region for species confirmation using Minimap2 [59, 60]. Phenotypic data pertaining to resistance against five different acaricide classes were available for the majority of the isolates, and these data were ascertained through LIT and LPT bioassays. From this pool of isolates, those displaying phenotypic resistance to at least one acaricide within each of the five classes, on the basis of the available LPT and LIT data, were chosen for further analysis. The phenotypic levels of resistance were categorized into three groups, determined by an RF calculated using a susceptible reference isolate [21, 61]. Isolates with an RF of less than 3 (RF < 3) were categorized as susceptible, those with an RF between 3 and 26 (3 ≤ RF ≤ 26) were classified as level I resistant, and those with an RF exceeding 26 (RF > 26) were considered level II resistant.

### DNA isolation

The tick preparation process for the chosen *R*. *microplus* isolates (*n* = 37) involved pooling 25 preserved larvae from each isolate, which were sterilely placed into 2 mL tubes filled with 20 μL of phosphate-buffered saline (PBS) and 200 μL of high-density zirconium oxide beads (2 × 2 mm diameter). The samples were homogenized for 2 × 30 second intervals and centrifuged at 7150 RCF for one minute. The lysate was processed via the Mammalian Tissue Genomic DNA Purification protocol and the GeneJET Genomic DNA Purification Kit (ThermoScientific). Digestion solution (180 μL) and proteinase K (20 μL) were added to each sample, which was subsequently centrifuged at 7150 RCF after vortexing. The samples were incubated at 56°C±2°C overnight. The tubes were subsequently centrifuged for one minute at 7150 RCF, and 20 μL of RNase A solution was added and incubated at room temperature for 10 minutes after vortexing each tube. Lysis solution (200 μL) was added to each tube, which was subsequently vortexed, centrifuged at 7150 RCF for one minute and incubated at room temperature for 10 minutes. Thereafter, 400 μL of 50% (v/v) ethanol was added to each tube, which was mixed by pulse vortexing and centrifuged at 7150 RCF for one minute. The cleared lysate was transferred to a spin column, and the extracted DNA was purified according to the manufacturer’s instructions. Spectrophotometric analysis was used to quantify the isolated DNA, and agarose gel electrophoresis was used to evaluate the integrity of the isolated DNA.

### Target identification and primer design

We selected exons encoding protein targets historically associated with tick acaricide resistance (Table 2) for sequencing. To achieve this goal, we used the Clinvet South Africa *R*. *microplus* genome (RmCVSA; GenBank accession number: JALIZG000000000) as a reference and mapped the coding sequences of each target to it. We used the Geneious plugin of Minimap2 with the long-read spliced alignment option to account for introns present in the reference genome. The genomic loci where the coding sequences mapped were annotated as exon regions. We then extracted and translated all these annotated regions to confirm their alignment with the encoded protein sequence of the target coding sequence.

**Table 2:** Targets implicated in tick acaricide resistance.

Target	Accession number
1. α-adrenergic-like octopamine receptor gene	JN974908
2. β-adrenergic-like octopamine receptor gene	JN974909
3. Octopamine–tyramine receptor gene	EF490687
4. GABA-gated chloride channel gene	GQ398111
5. Glutamate-gated chloride channel gene	KF881800
6. Acetylcholine esterase 2	AJ278345
7. Acetylcholine esterase 3	GU944960
8. Voltage-gated sodium channel gene	AF134216 (partial)
9. Carboxylesterase gene	DQ533868
10. Cytochrome P450 CYP4W1	AF081807
11. Cytochrome P450 CYP41	HM012801

We designed oligonucleotide primers in intronic regions flanking the exon(s) of interest to ensure that the native exon sequences were obtained during sequencing. Our primer design targeted multiple exons per amplicon while keeping the PCR product size under 3,500 bp. This strategy accommodated partially degraded template DNA and facilitated efficient PCR amplification.

### Target amplification and sequencing

#### PCR amplification of targets and purification

Approximately 10 ng of DNA was used as a template in a reaction volume of 10 μL with 4 μL of each multiplex primer mixture (concentrations in S2 Table) and 5 μL of Platinum™ SuperFi II DNA Polymerase (Invitrogen). The reaction mixtures were subjected to the following thermal cycling conditions: initial denaturation for 2 minutes at 98°C, followed by 30 cycles at 98°C for 10 seconds, 60°C for 20 seconds, and 72°C for 2 minutes, with a final elongation stage of 5 minutes at 72°C and a hold at 4°C. The PCR amplicons were visualized through agarose gel electrophoresis. This process involved the use of a 2% (m/v) agarose gel in 1x TAE buffer, which contained a 1x SYBR Safe stain. Electrophoresis was conducted at 6 V/cm for 50 minutes. The PCR products of all the targets were pooled (per isolate) and subjected to DNA purification using the AMPure XP beads (Beckman Coulter) according to the manufacturer’s recommendations. Purified DNA was quantified using the Qubit™ 1X dsDNA Broad Range (BR) assay kit and was used as input material for our custom end preparation protocol. The SQK-LSK109 ligation sequencing kit was used for amplicon library preparation, with the following modifications. Briefly, 200 fmol of PCR product (based on an average amplicon size of 1.5 kb) was mixed with 1 μl of DNA control sample (DCS), 6 μl of PNK Buffer A, 6 μl of 10 mM ATP, 3 μl of 10 mM dATP, 2.5 units of Thermopol Taq Polymerase, and 10 units of PNK in a final volume of 64.5 μl and incubated for 30 minutes at 37°C, followed by incubation at 65°C for 30 minutes. The prepared amplicons were then subjected to clean-up with AMPure XP beads (Beckman Coulter) according to the manufacturer’s recommendations and eluted with 61 μL of 5 mM Tris, pH 8.5.

The purified sample was then subjected to adapter ligation by mixing 30 μl of the sample with 12.5 μl of Ligation Buffer (LNB), 2.5 μl of Adapter Mix (AMX), and 2000 units of NEB T4 DNA ligase and incubating the mixture for 20 minutes at room temperature. Subsequently, 40 μl AMPure XP beads were added to the mixture and incubated for 5 minutes, after which the sample was allowed to pellet on the magnetic separation rack. The supernatant was removed with a pipette, and the beads were washed twice with short fragment buffer (SFB), eluted with 15 μL of elution buffer (EB), and quantified via the Qubit™ 1X dsDNA High Sensitivity Assay Kit.

#### Sequencing

The DNA library was prepared by mixing 50 fmol (based on an average DNA amplicon size of 1). 5 kb) of the library with sequencing buffer (SQB) and loading beads (LB). Subsequently, 75 μL of the DNA library was loaded into the MinION R9.4.1 flow cell and sequenced for approximately 16 hours to produce Fast5 files.

The Fast5 files produced by the MinION device for all the target amplicons (per isolate) were subjected to GPU guppy base calling (version 5.0.11) using the super high accuracy (SUP) model and the dna_r9.4.1_450 bps_sup.cfg configuration file (filtering parameter—min_qscore 10). The resulting sequence reads (in fastq format) for each isolate were concatenated and converted with seqtk to fasta format and mapped to the target reference genomes using Minimap2 using default [59, 60].

Specifically, the combined FASTA file for each isolate was mapped to one target reference, which produced a sequence alignment map (SAM) file. SAMtools [62] was used to extract the unused reads from this alignment, and these unmapped reads were aligned to the next target. This was performed recursively until the reads were mapped to all the target reference genomes.

The alignments in SAM format were imported into Geneious Prime (version 2022.2.2) and inspected to ensure that sufficient exon coverage was achieved across all the targets for all the isolates. Thereafter, consensus sequences (75% threshold) were generated for all the alignments in Geneious. The introns were removed from the consensus sequences for all the isolates, and the resulting coding regions were multiple aligned per target using MAFFT (version 7.490) [63]. Non-synonymous mutations in the multiple sequence alignments were analysed for each target and compared between the acaricide-susceptible and -resistant isolates. We also examined whether previously reported non-synonymous mutations were present in our isolates.

### Three-dimensional modelling and ligand docking

Amino acid substitutions, which were more prevalent in resistant isolates than in susceptible isolates, were subjected to 3D modelling and ligand docking analysis. The AlphaFold database [64] was used to retrieve 3D structure models (pdb format) for all the target proteins in this study. Acaricide/metabolite 3D structures (sdf format) were obtained from PubChem. We utilized CB-Dock for blind docking of the protein and the 3D ligand structures, assessing a total of 16 potential cavities for docking evaluation [65]. Docking was only performed when no structural information (based on the literature) regarding the ligand binding site was available. The *R*. *microplus* VGSC protein 3D homology model was generated via SWISS-MODEL [66], which employs the *Periplaneta americana* (American cockroach VGSC cryo-EM structure with a resolution of 2.8 Å (PDB: 6a90)) as a template [67]. YASARA View was used to visualize and annotate the residues of interest [68].

## Supporting information

S1 Fig Amino acid substitutions in βAOR gene and 3D structure in *R*. *microplus* isolates. (TIF)

S2 Fig The *R*. *microplus* βAOR binding pocket. (TIF)

S3 Fig Amino acid substitutions in αAOR gene and 3D structure in *R*. *microplus* isolates. (TIF)

S4 Fig Amino acid substitutions in OCT/TYR gene in *R*. *microplus* isolates. (TIF)

S5 Fig AlphaFold 3D structure of the *R*. *microplus* OCT/TYR receptor. (TIF)

S6 Fig Amino acid substitutions in GABA-Cl gene and 3D structure in *R*. *microplus* isolates. (TIF)

S7 Fig Amino acid substitutions in the AChE2 gene in *R*. *microplus* isolates. (TIF)

S8 Fig AlphaFold 3D structure of *R*. *microplus* AChE2. (TIF)

S9 Fig Multiple sequence alignment of amino acid substitutions in AChE3 in *R*. *microplus* isolates. (TIF)

S10 Fig AlphaFold 3D structure of *R*. *microplus* AChE3. (TIF)

S11 Fig Multiple sequence alignment of substitutions observed in the CBE gene from *R*. *microplus* isolates. (TIF)

S12 Fig Multiple sequence alignment of mutations observed in the CBE gene in *R*. *microplus* isolates. (TIF)

S13 Fig The 3D structure of *R*. *microplus* CBE with mutations and deltamethrin docking. (TIF)

S14 Fig Amino acid substitutions in CYP4W1 gene and 3D structure in *R*. *microplus* isolates. (TIF)

S15 Fig Multiple sequence alignment of substitutions observed in the CYP4W1 in *R*. *microplus* isolates. (TIF)

S16 Fig Multiple sequence alignment of mutations observed in the CYP41 gene in *R*. *microplus* isolates. (TIF)

S17 Fig Multiple sequence alignment of mutations observed in the CYP41 gene in *R*. *microplus* isolates. (TIF)

S18 Fig AlphaFold 3D structure of *R*. *microplus* CYP41. (TIF)

S1 Table *Rhipicephalus microplus* isolates included in this study and the test used to determine the phenotypic resistance to different acaricides. (DOCX)

S2 Table List of multiplex PCR oligonucleotides used to amplify *R*. *microplus* targets. (DOCX)

S3 Table Amino acid substitutions in acaricide target genes in *R*. *microplus* isolates from this study. (DOCX)
