# Supplementary figures and images for: Genomic assessment of targets implicated in Rhipicephalus microplus acaricide resistance
Source: PLoS One. 2024 Dec 5;19(12):e0312074. doi: 10.1371/journal.pone.0312074 (PMC11620669; doi:10.1371/journal.pone.0312074)

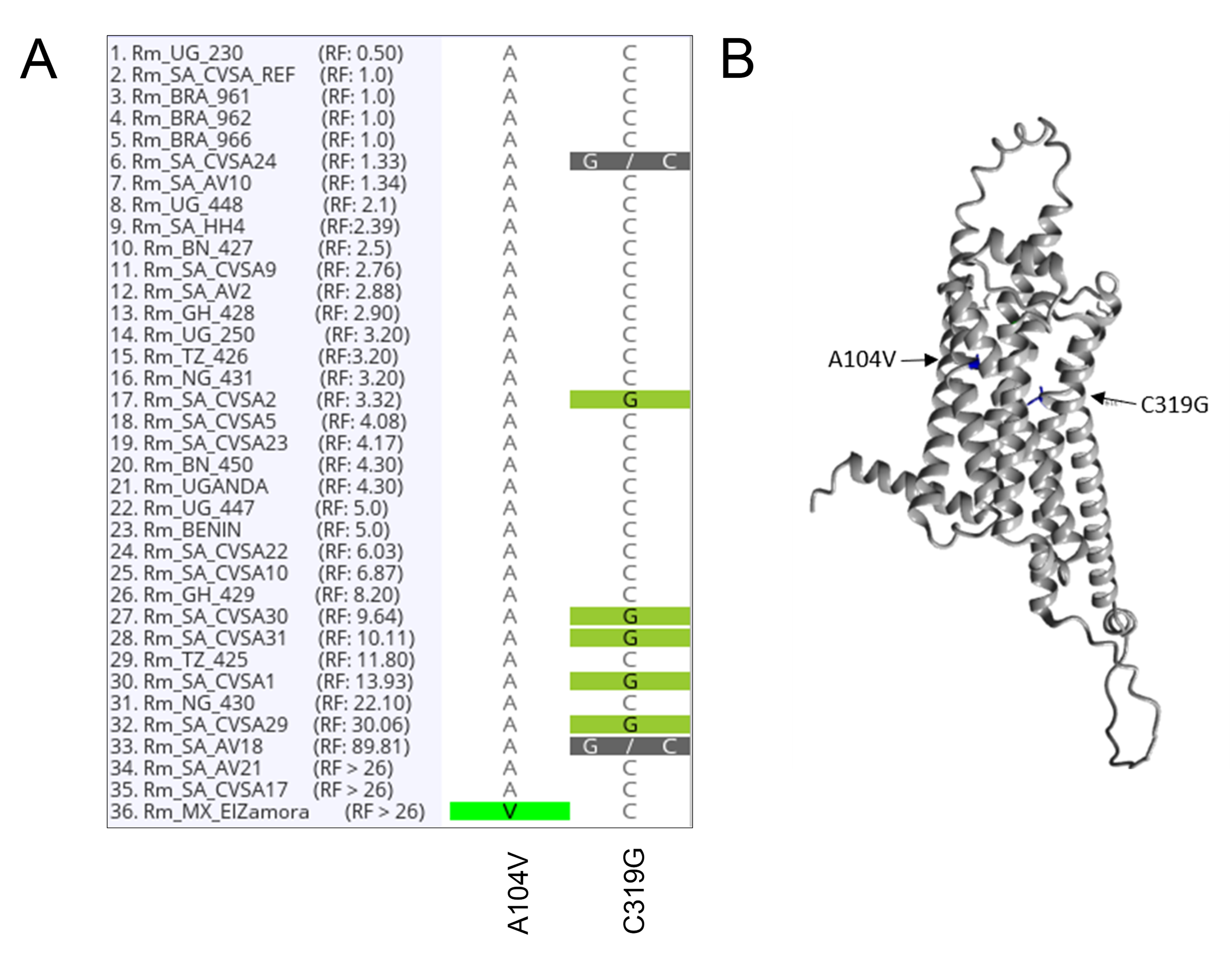

Supplement: S1 Fig — (TIF) [file pone.0312074.s001.tif]

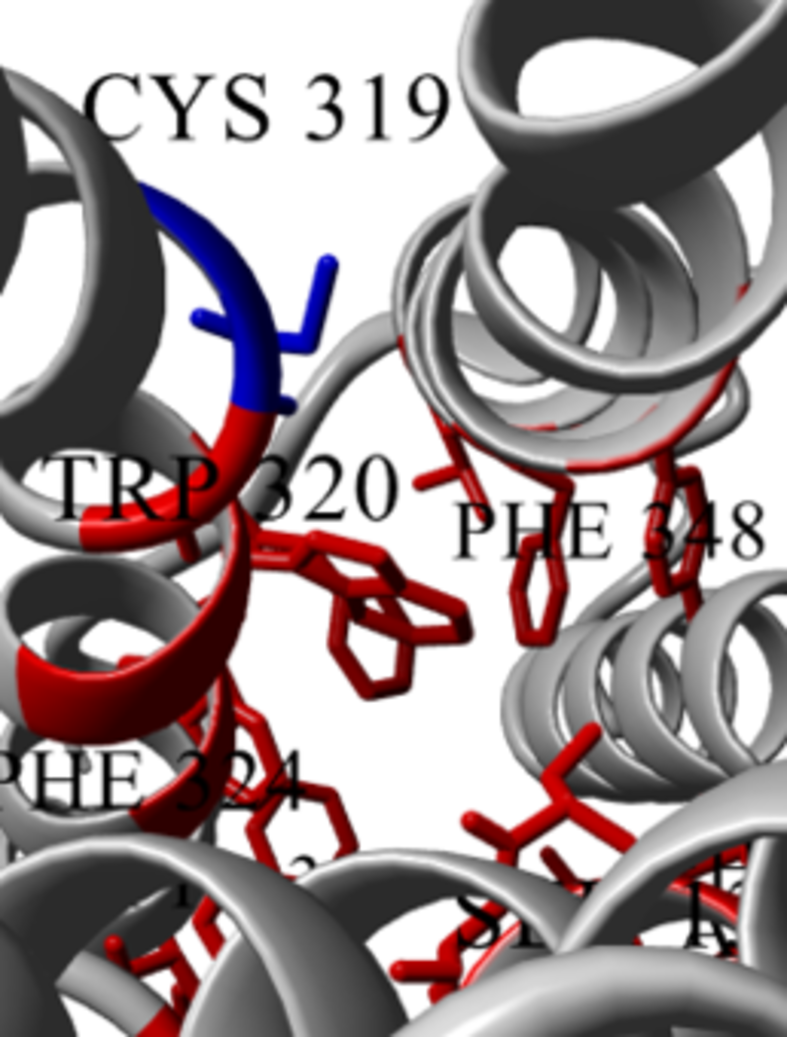

Supplement: S2 Fig — (TIF) [file pone.0312074.s002.tif]

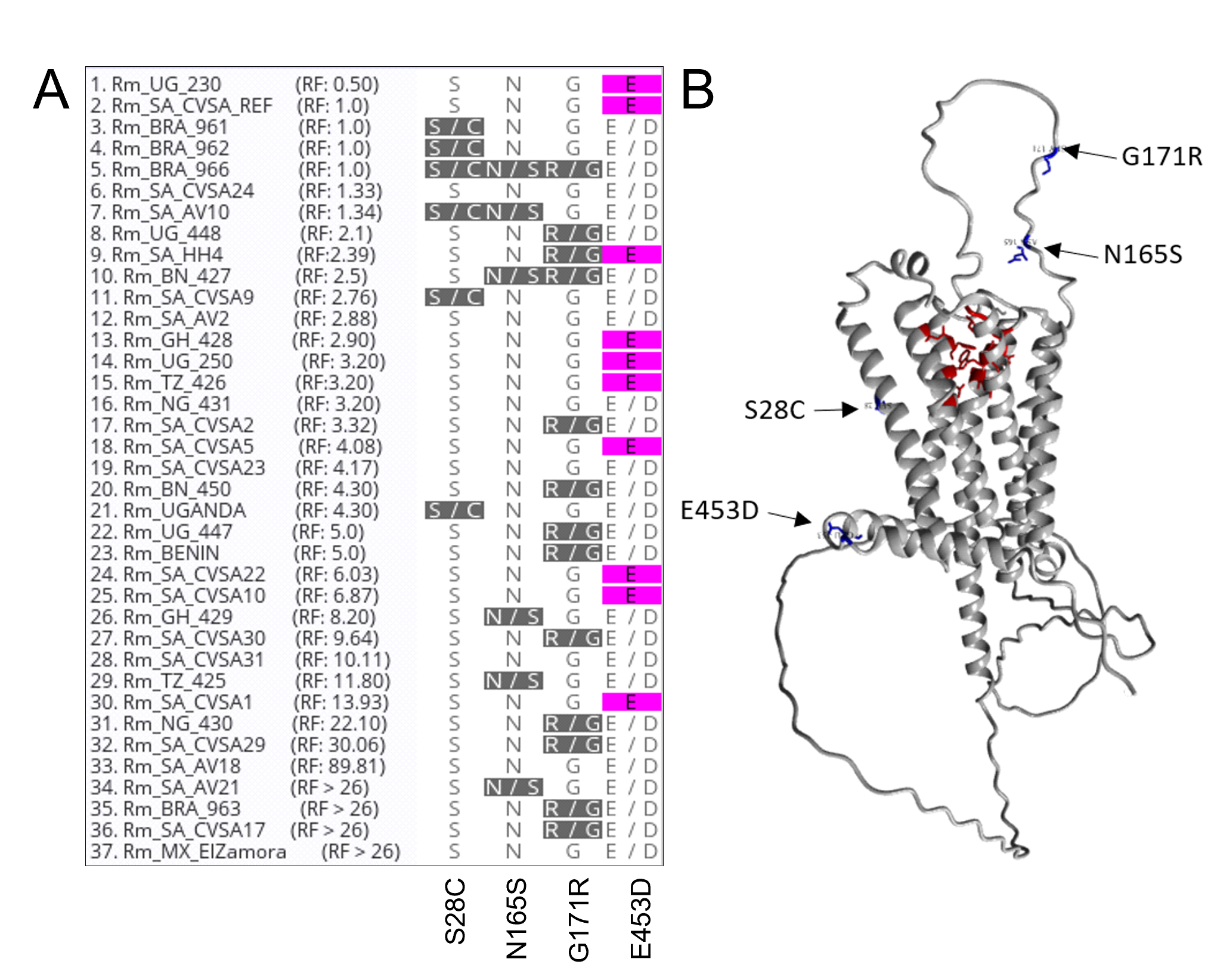

Supplement: S3 Fig — (TIF) [file pone.0312074.s003.tif]

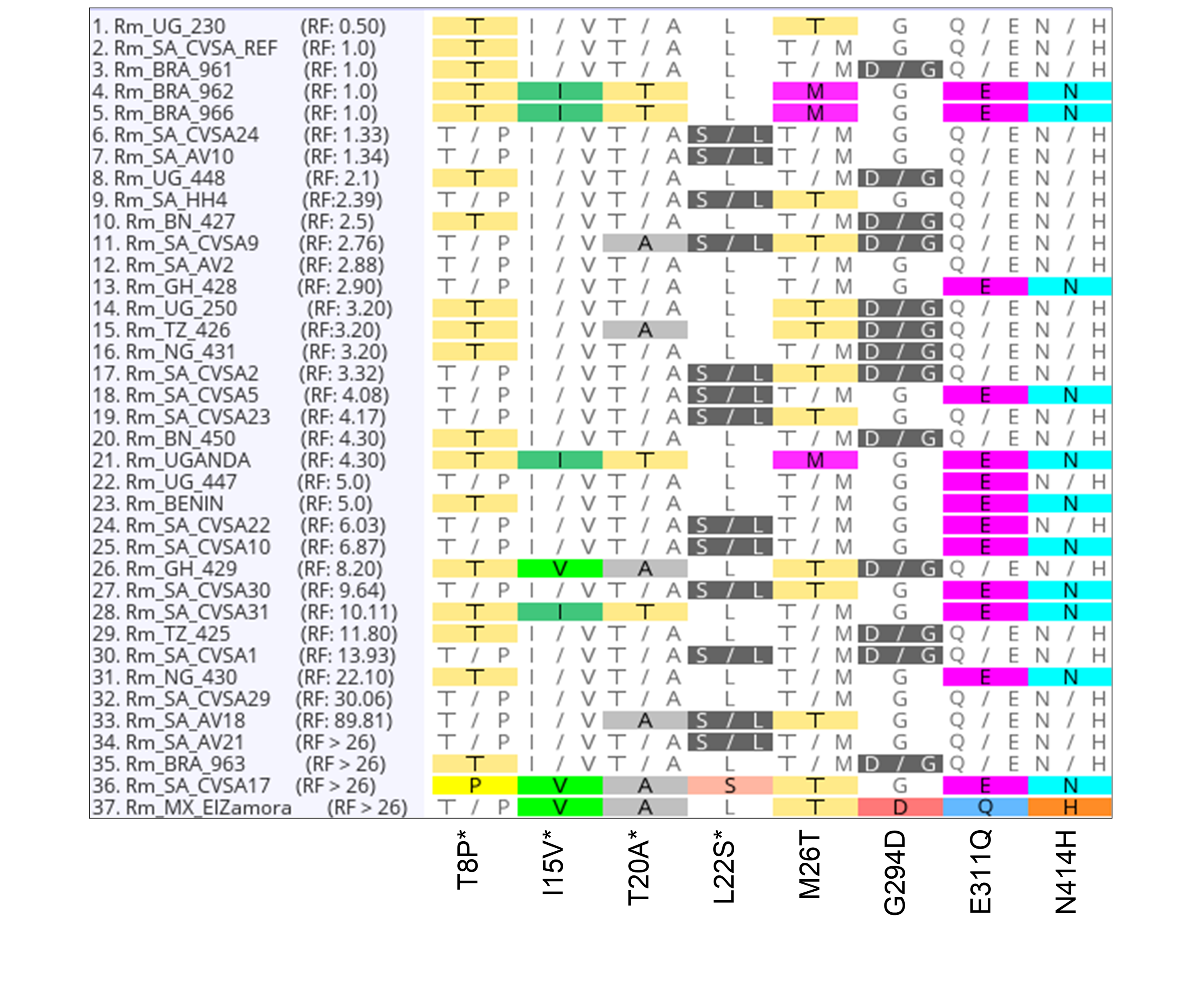

Supplement: S4 Fig — (TIF) [file pone.0312074.s004.tif]

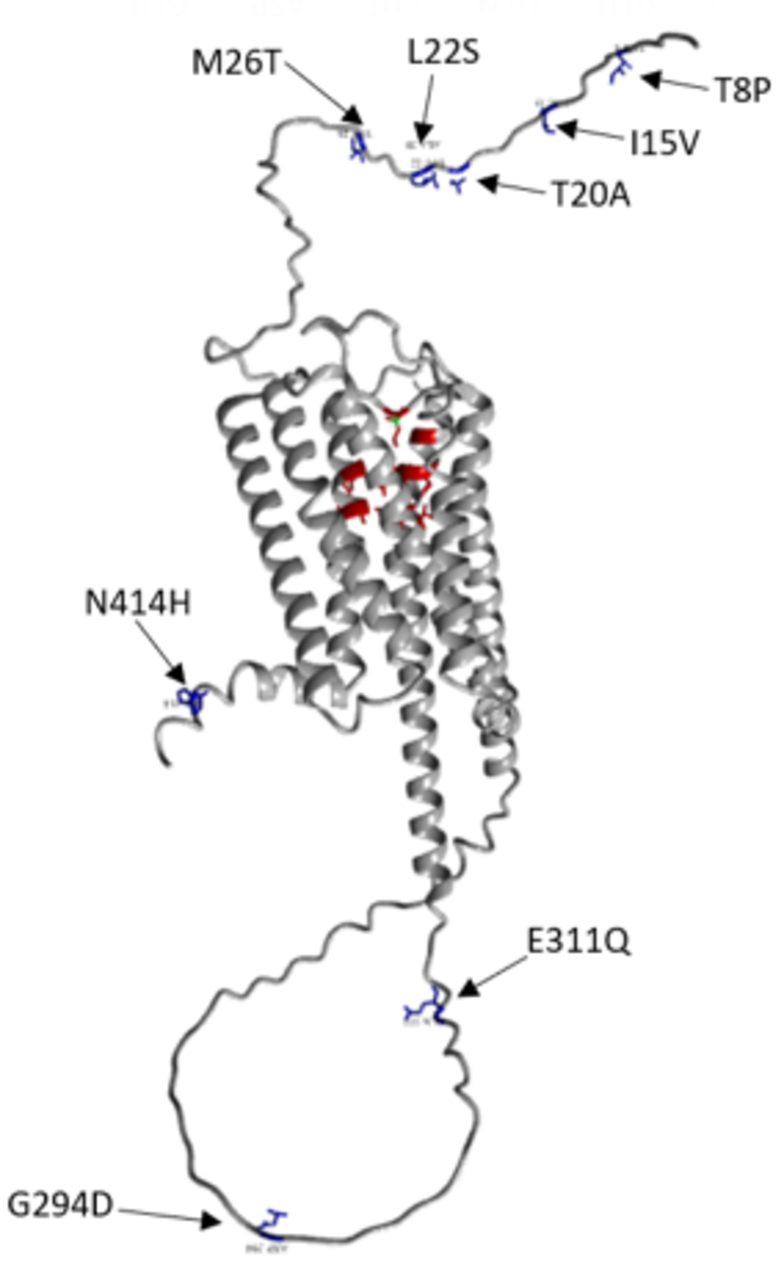

Supplement: S5 Fig — (TIF) [file pone.0312074.s005.tif]

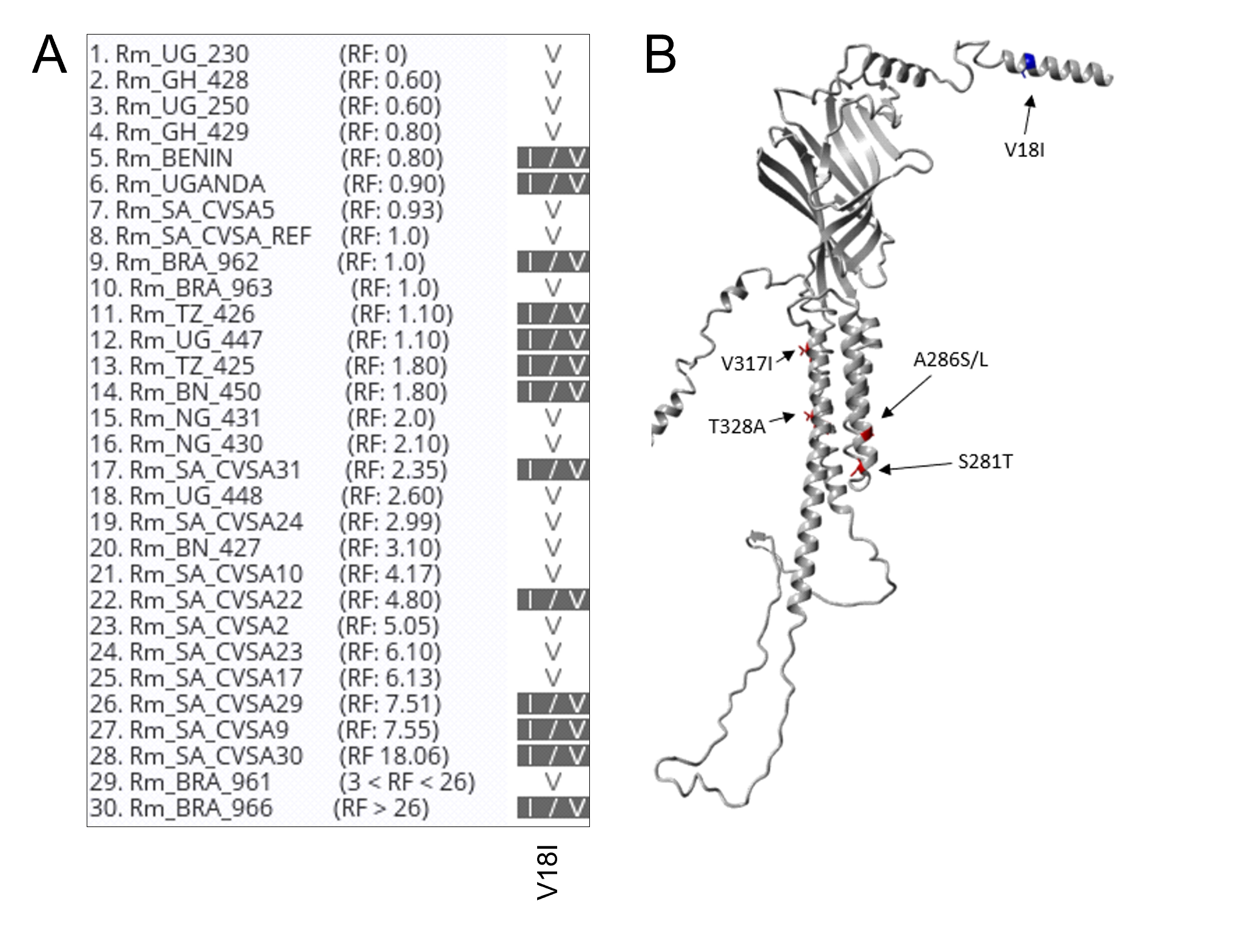

Supplement: S6 Fig — (TIF) [file pone.0312074.s006.tif]

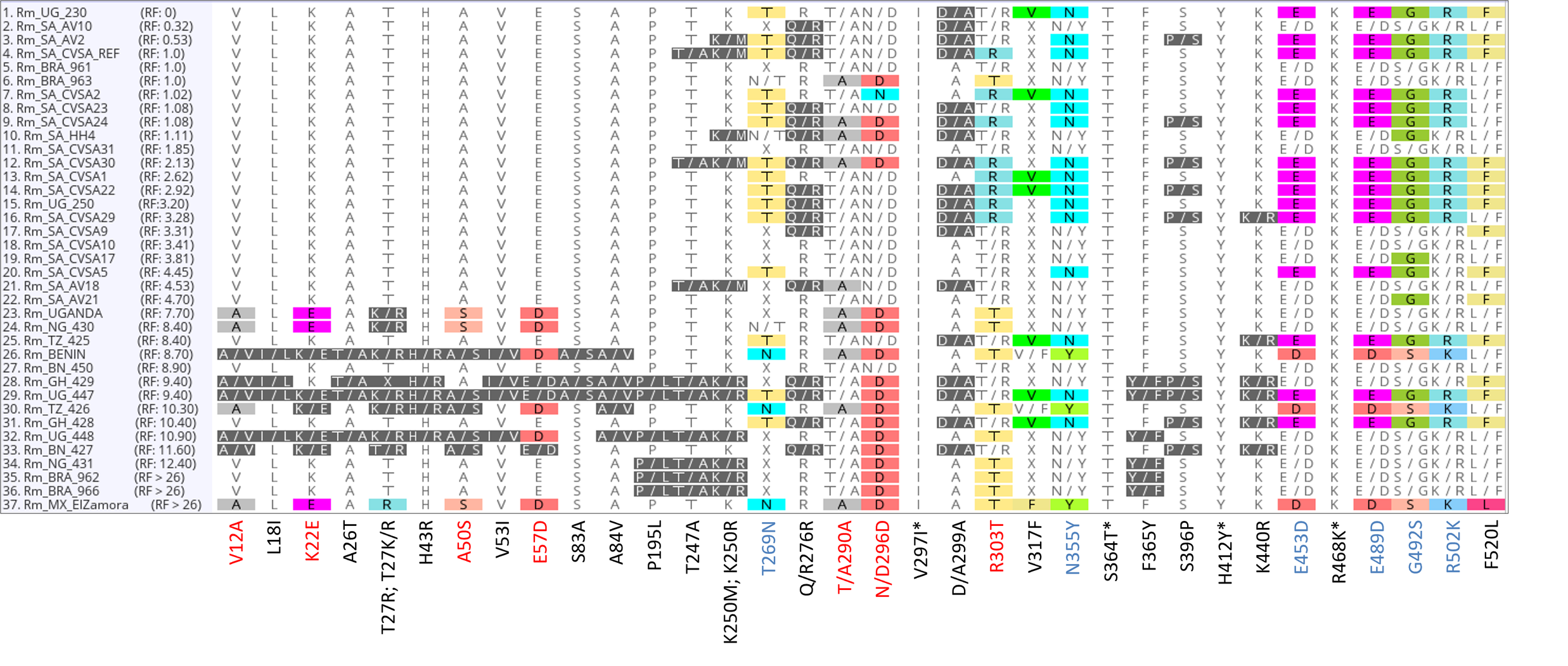

Supplement: S7 Fig — (TIF) [file pone.0312074.s007.tif]

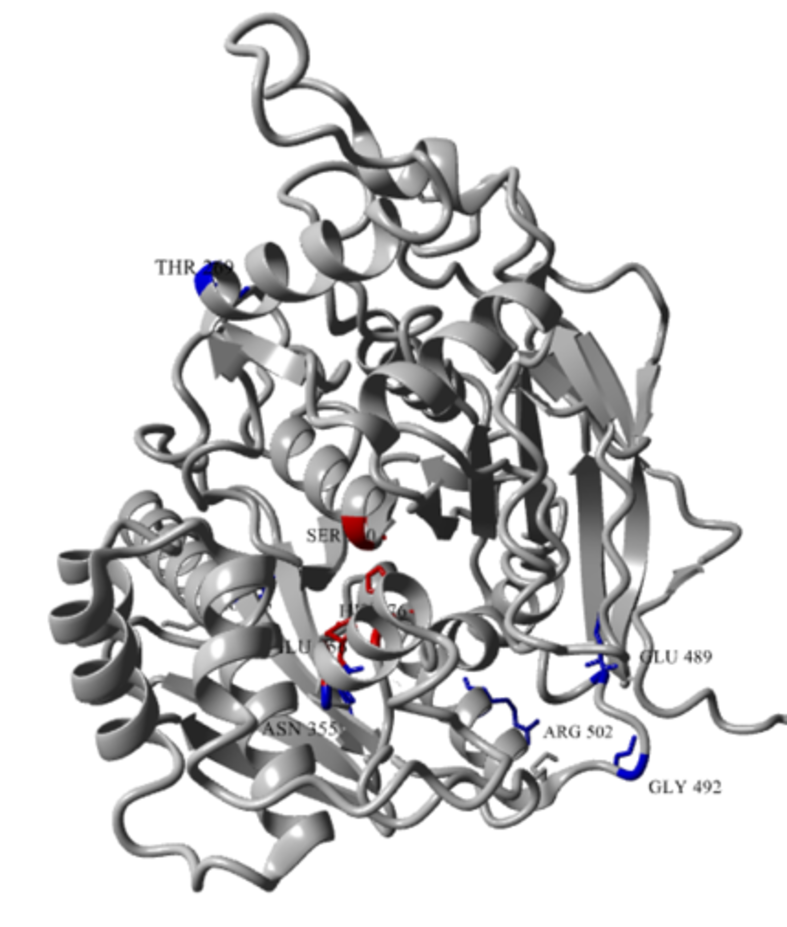

Supplement: S8 Fig — (TIF) [file pone.0312074.s008.tif]

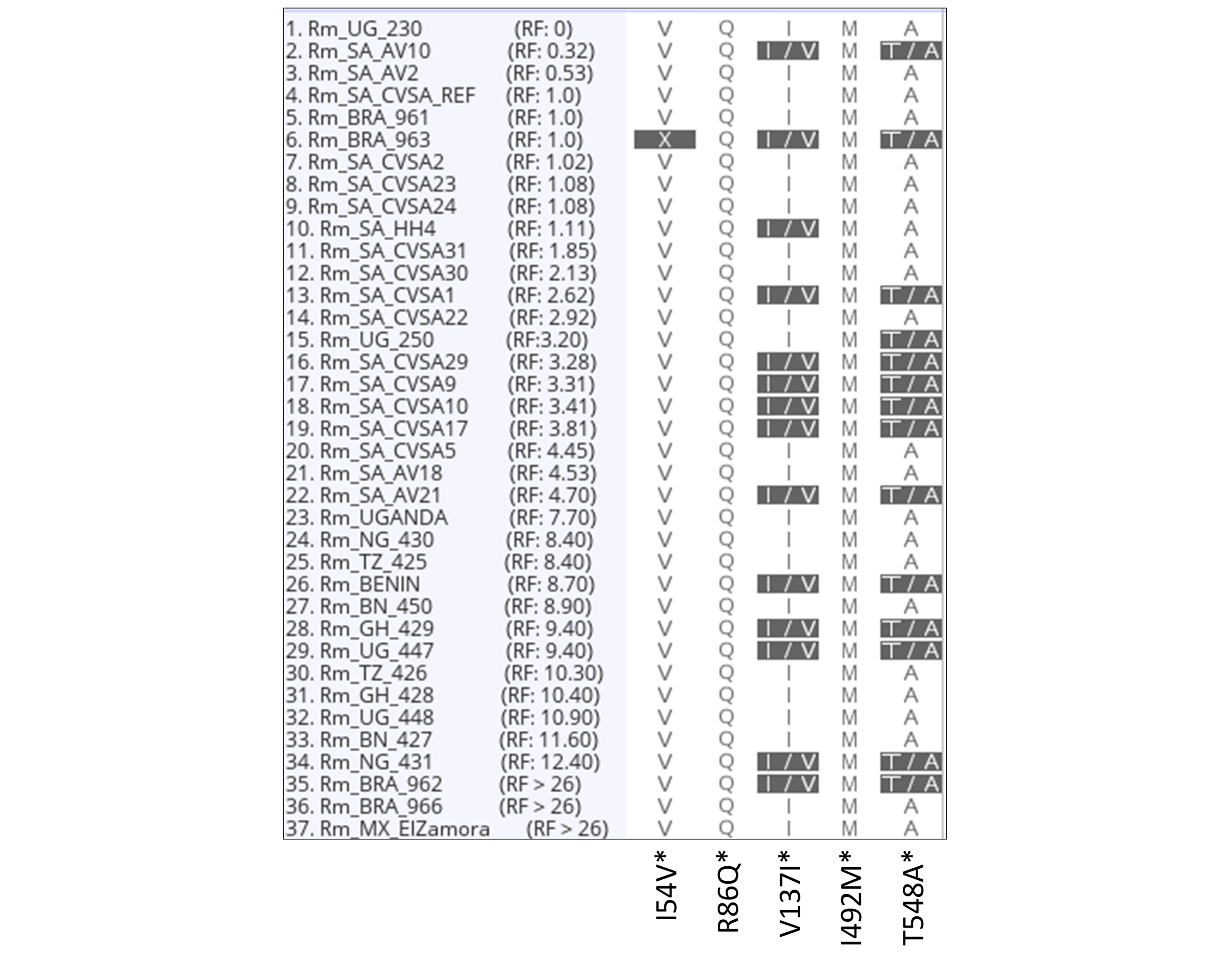

Supplement: S9 Fig — (TIF) [file pone.0312074.s009.tif]

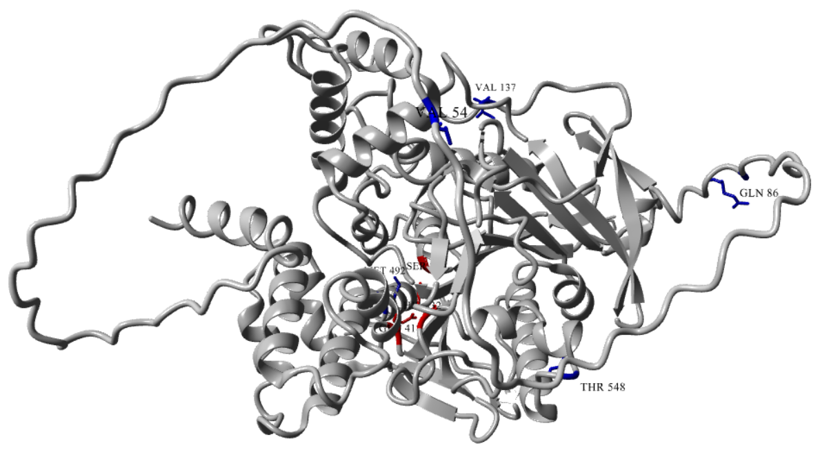

Supplement: S10 Fig — (TIF) [file pone.0312074.s010.tif]

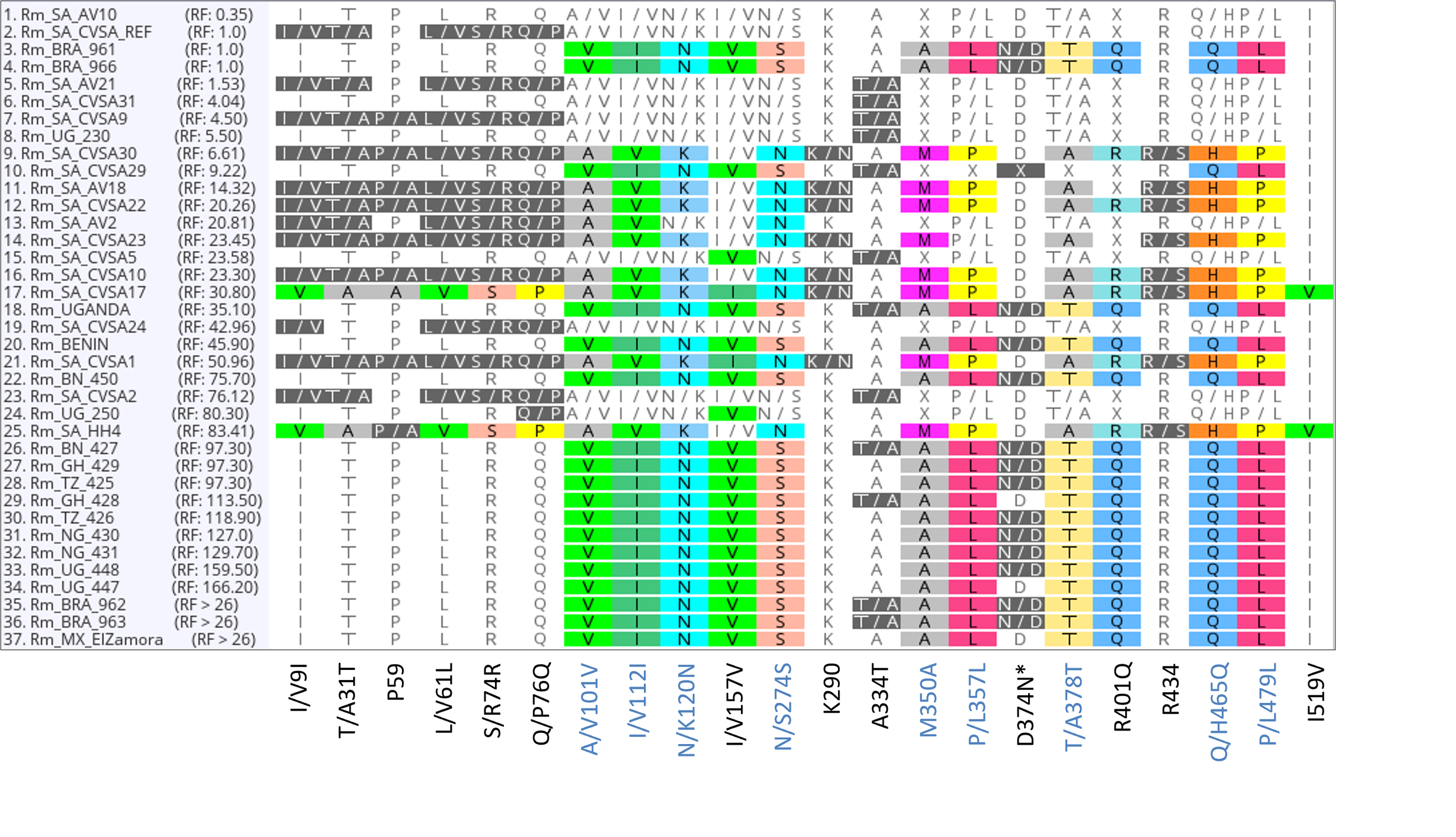

Supplement: S11 Fig — (TIF) [file pone.0312074.s011.tif]

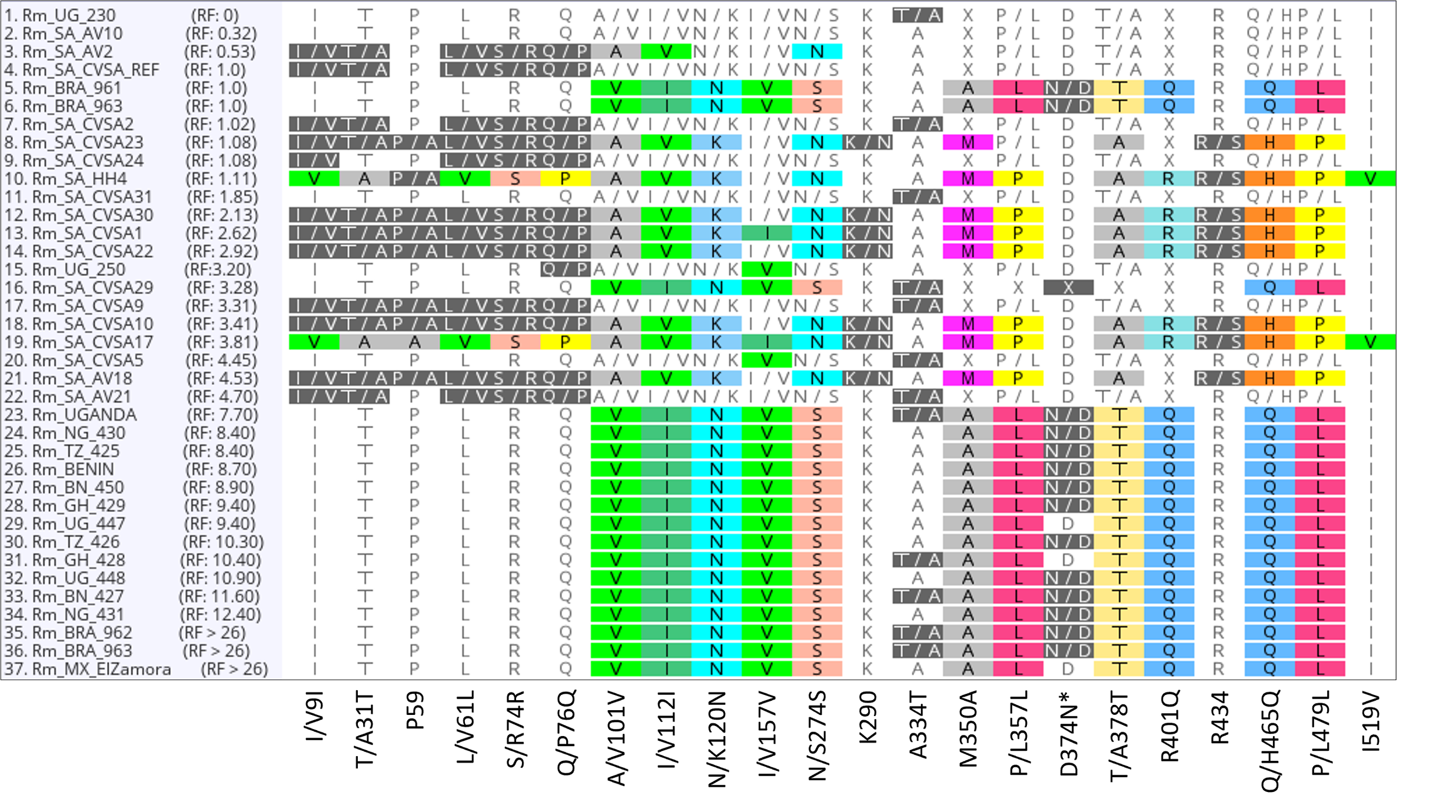

Supplement: S12 Fig — (TIF) [file pone.0312074.s012.tif]

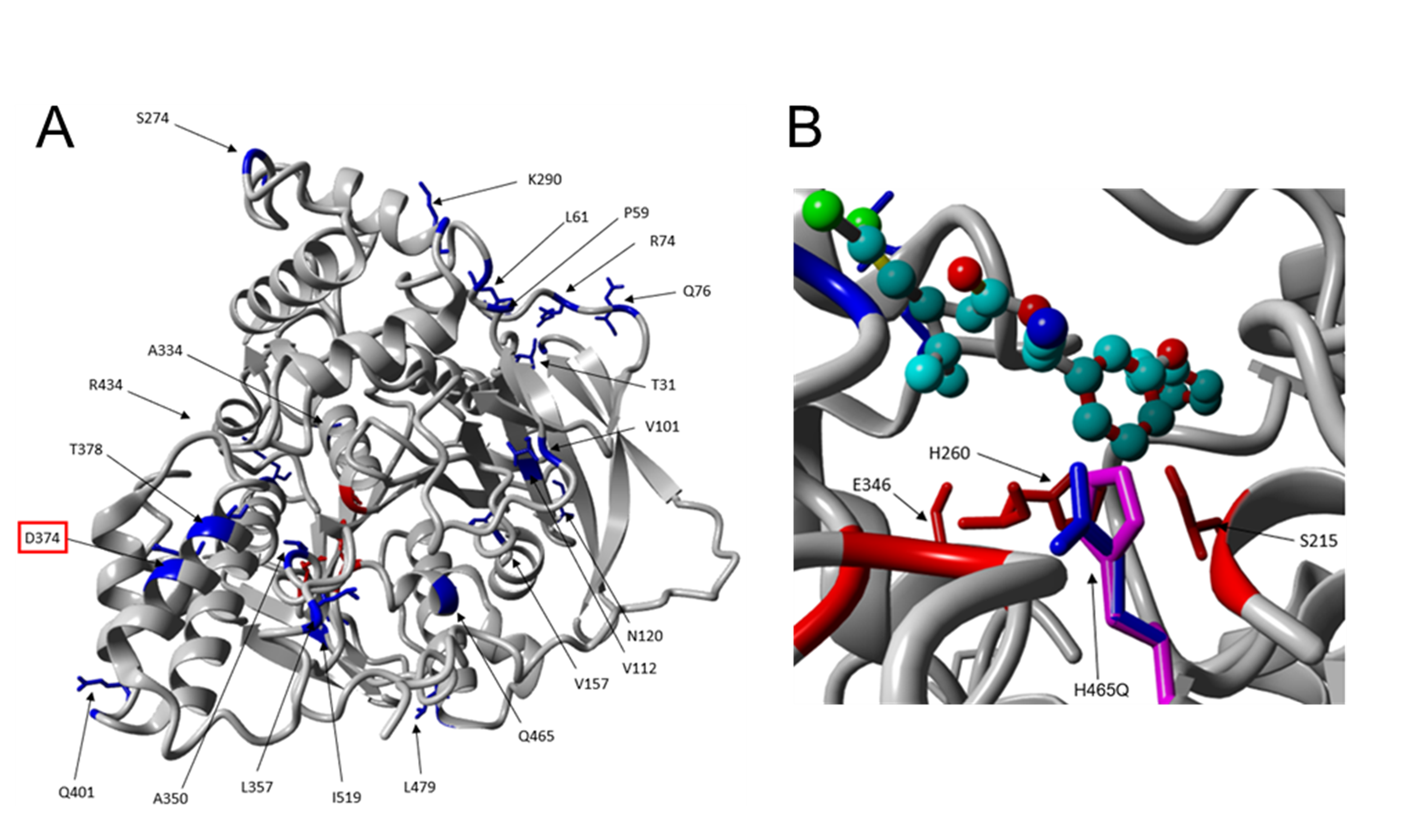

Supplement: S13 Fig — (TIF) [file pone.0312074.s013.tif]

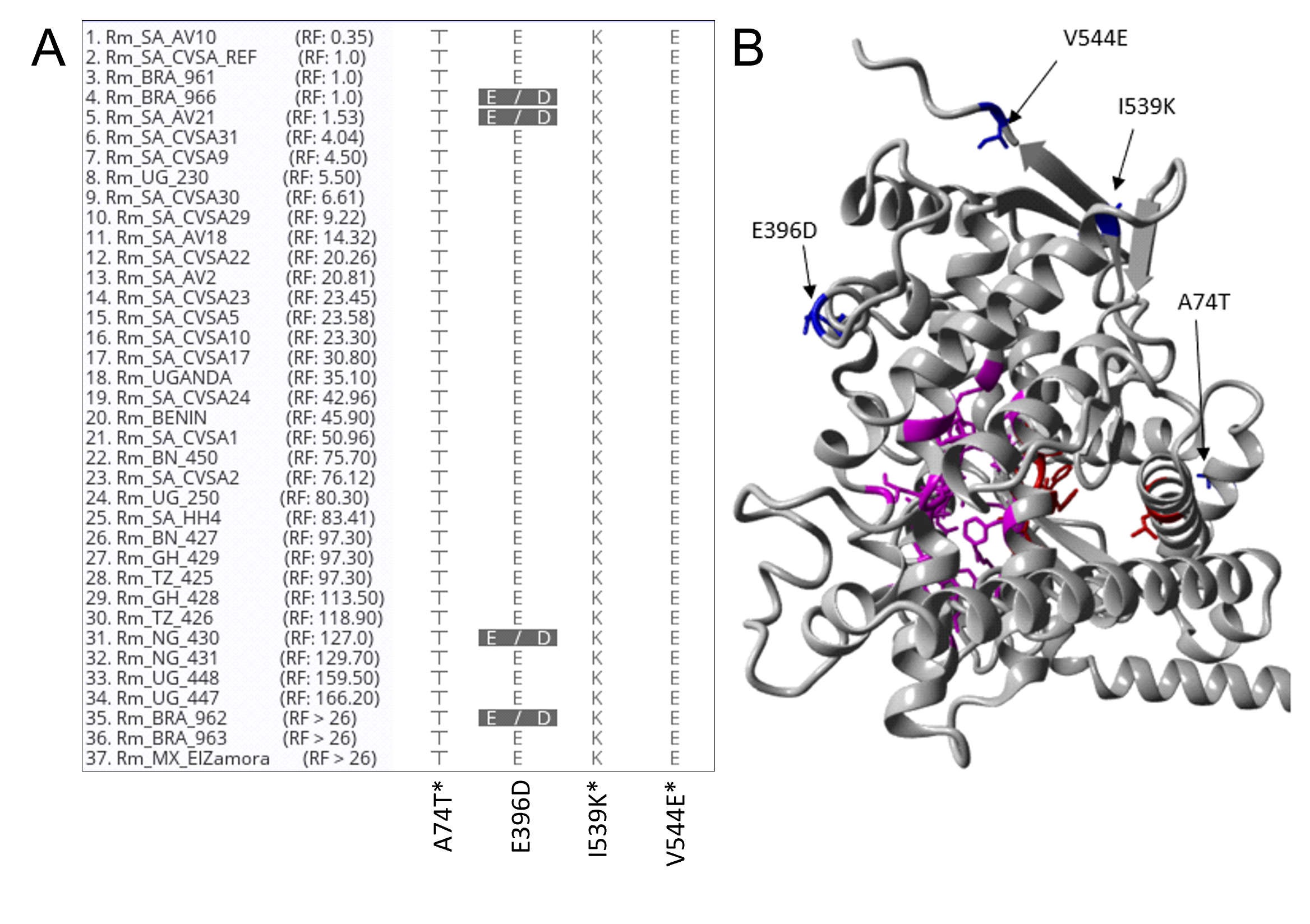

Supplement: S14 Fig — (TIF) [file pone.0312074.s014.tif]

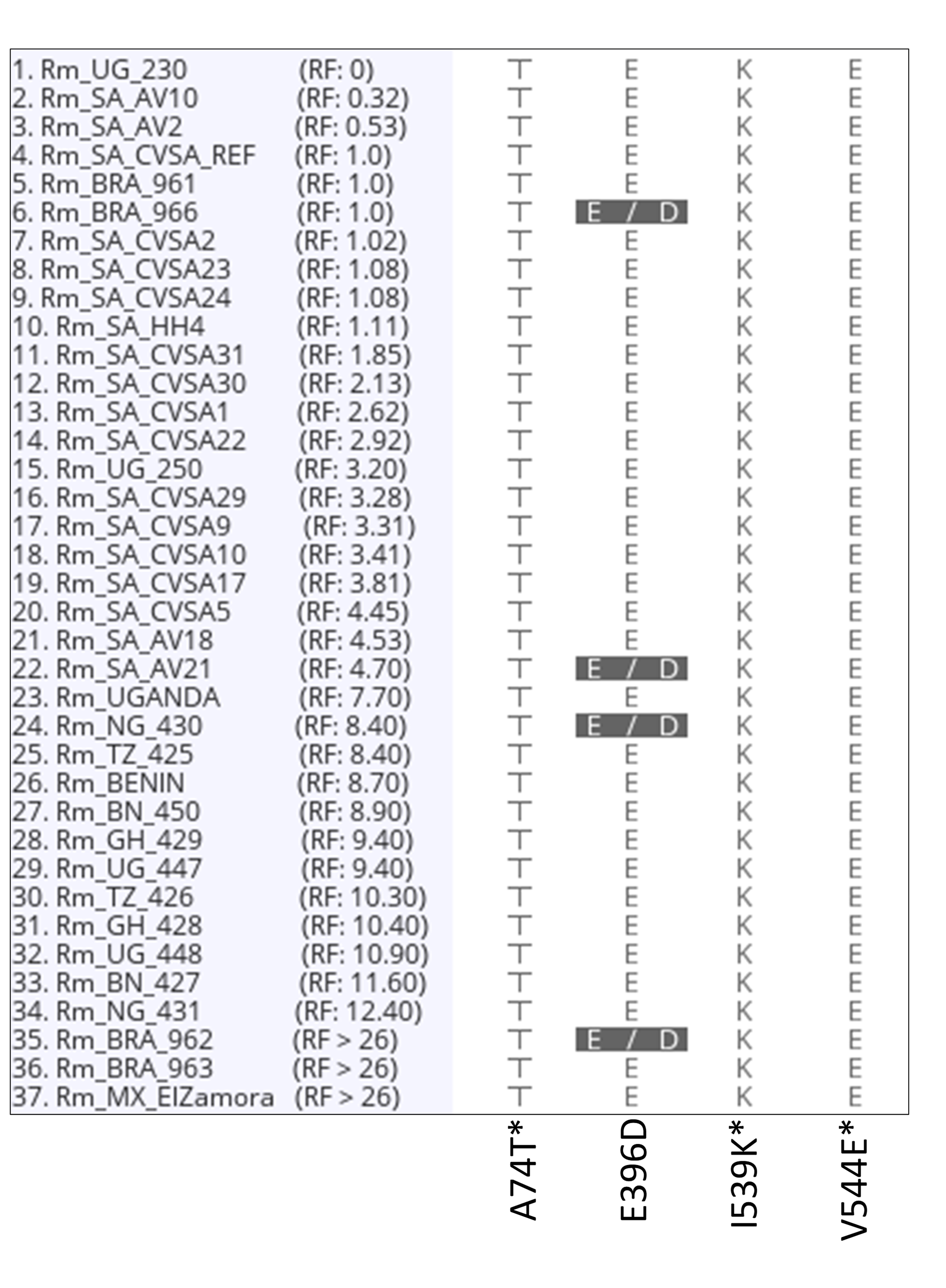

Supplement: S15 Fig — (TIF) [file pone.0312074.s015.tif]

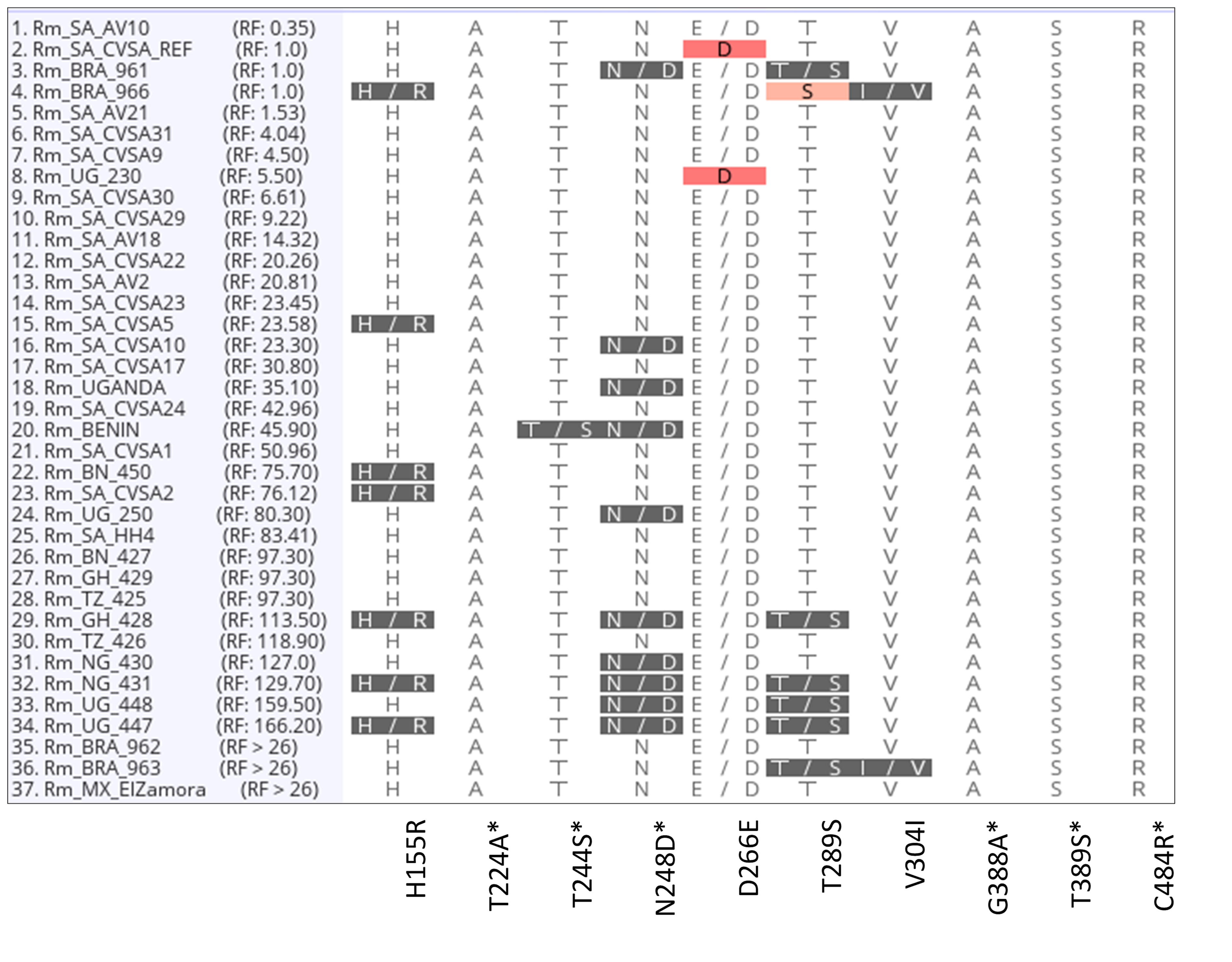

Supplement: S16 Fig — (TIF) [file pone.0312074.s016.tif]

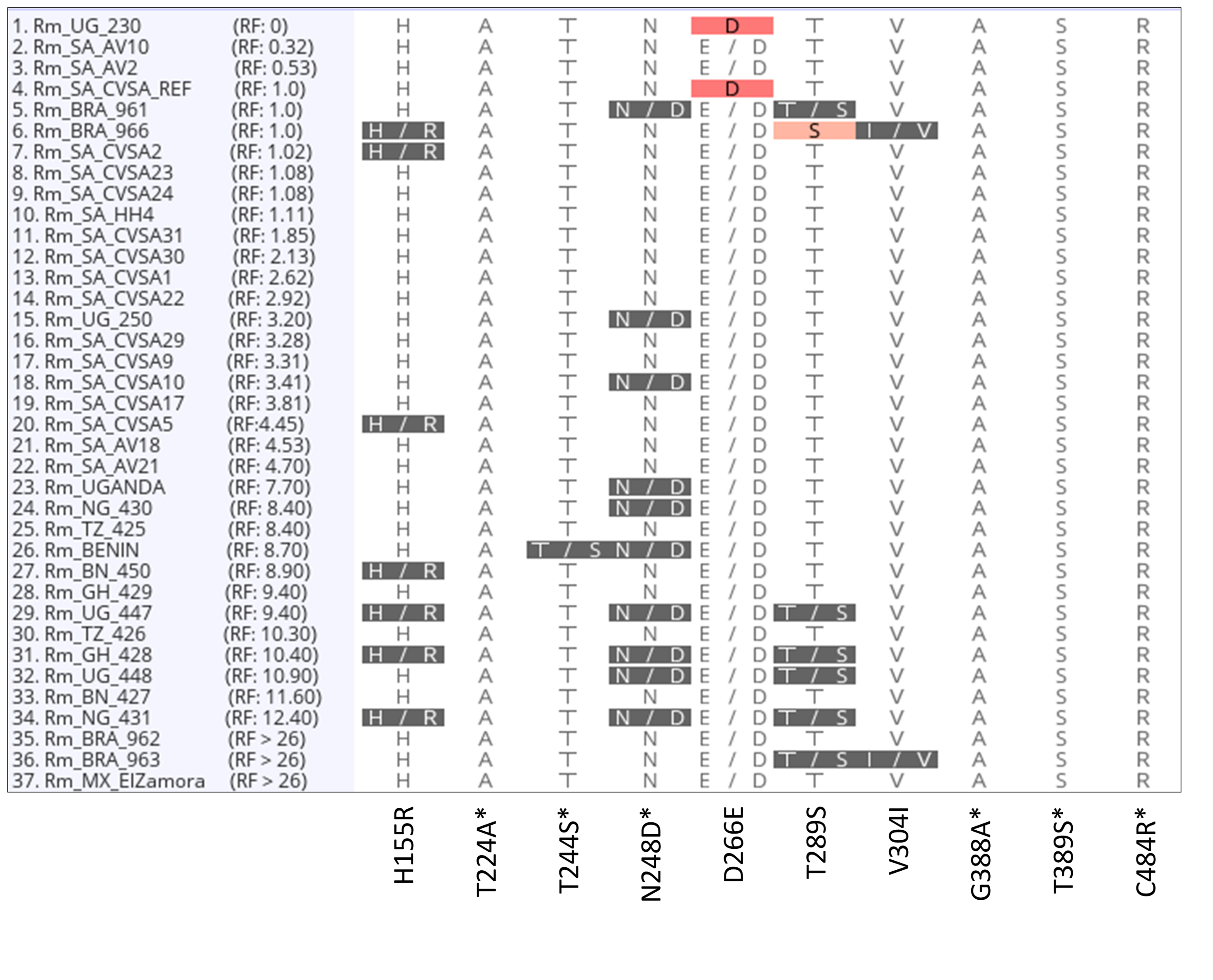

Supplement: S17 Fig — (TIF) [file pone.0312074.s017.tif]

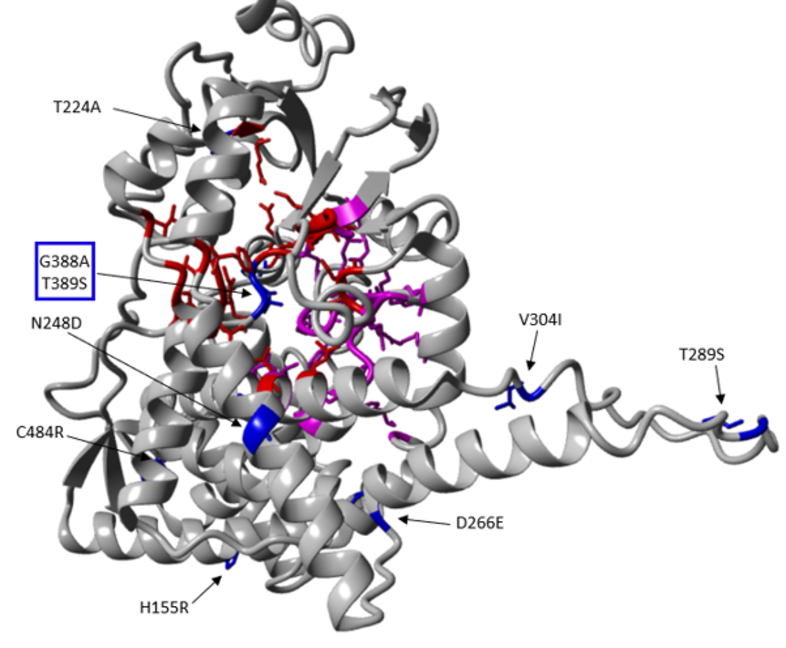

Supplement: S18 Fig — (TIF) [file pone.0312074.s018.tif]
