# Supplementary material for: Genomic assessment of targets implicated in Rhipicephalus microplus acaricide resistance
Source: PLoS One. 2024 Dec 5;19(12):e0312074. doi: 10.1371/journal.pone.0312074 (PMC11620669; doi:10.1371/journal.pone.0312074)
Supplement: S1 Table — (DOCX) [file pone.0312074.s019.docx]

Table S1. *Rhipicephalus microplus* isolates included in this study and the test used to determine the phenotypic resistance to different acaricides.

**Isolate Alternative Sampling Bioassay Acaricides tested**

**ID ID location with the bioassay**

1. Rm_SA_AV2 CG0002 South Africa LPT Amitraz,

chlorphenvinpos,

deltamethrin

1. Rm_SA_AV10 CG010 South Africa LPT Amitraz,

chlorphenvinpos,

deltamethrin

1. Rm_SA_AV18 CG018 South Africa LPT Amitraz,

chlorphenvinpos,

deltamethrin

1. Rm_SA_AV21 CG021 South Africa LPT Amitraz,

chlorphenvinpos,

deltamethrin

1. Rm_SA_CVSA1 CVSA0001 South Africa LPT Amitraz,

chlorphenvinpos,

deltamethrin,

doramectin

1. Rm_SA_CVSA2 CVSA0002 South Africa LPT Amitraz,

chlorphenvinpos,

deltamethrin,

doramectin,

fipronil

1. Rm_SA_CVSA5 CVSA0005 South Africa LPT Amitraz,

chlorfenvinphos,

deltamethrin, fipronil

1. Rm_SA_CVSA9 CVSA0009 South Africa LPT Amitraz,

chlorphenvinpos,

deltamethrin,

doramectin,

fipronil

1. Rm_SA_CVSA10 CVSA0010 South Africa LPT Amitraz,

chlorphenvinpos,

deltamethrin,

doramectin,

fipronil

1. Rm_SA_CVSA17 CVSA0017 South Africa LPT Amitraz,

chlorphenvinpos,

deltamethrin,

doramectin,

fipronil

1. Rm_SA_CVSA22 CVSA0022 South Africa LPT Amitraz,

chlorphenvinpos,

deltamethrin,

doramectin,

fipronil

1. Rm_SA_CVSA23 CVSA0023 South Africa LPT Amitraz,

chlorphenvinpos,

deltamethrin,

doramectin,

fipronil

1. Rm_SA_CVSA24 CVSA0024 South Africa LPT Amitraz,

chlorphenvinpos,

deltamethrin,

doramectin,

fipronil

1. Rm_SA_CVSA29 CVSA0029 South Africa LPT Amitraz,

chlorphenvinpos,

deltamethrin,

doramectin,

fipronil

1. Rm_SA_CVSA30 CVSA0030 South Africa LPT Amitraz,

chlorphenvinpos,

deltamethrin,

doramectin,

fipronil

1. Rm_SA_CVSA31 CVSA0031 South Africa LPT Amitraz,

chlorphenvinpos,

deltamethrin,

doramectin,

fipronil

1. Rm_SA_HH4 HH0004 South Africa LPT Amitraz,

chlorfenvinphos,

deltamethrin

1. Rm_SA_CVSA_REF CVSA South Africa LPT Amitraz,

chlorphenvinpos,

deltamethrin,

doramectin,

fipronil

1. Rm_BRA_961 D21-00961 Brazil LIT Amitraz,

chlorphenvinpos,

alpha-cypermethrin,

ivermectin, fipronil

1. Rm_BRA_962 D21-00962 Brazil LIT Amitraz,

chlorphenvinpos,

alpha-cypermethrin,

ivermectin, fipronil

1. Rm_BRA_963 D21-00963 Brazil LIT Amitraz,

chlorphenvinpos,

alpha-cypermethrin,

ivermectin, fipronil

1. Rm_BRA_966 D21-00966 Brazil LIT Amitraz,

chlorphenvinpos,

alpha-cypermethrin,

ivermectin, fipronil

1. Rm_TZ_425 D19-01432 Tanzania LPT Amitraz,

chlorphenvinpos,

alpha-cypermethrin,

ivermectin, fipronil

1. Rm_TZ_426 D19-01426 Tanzania LPT Amitraz,

chlorphenvinpos,

alpha-cypermethrin,

ivermectin, fipronil

1. Rm_BN_427 D19-01427 Benin LPT Amitraz,

chlorphenvinpos,

alpha-cypermethrin,

ivermectin, fipronil

1. Rm_BENIN Rm_Benin Benin LPT Amitraz,

chlorphenvinpos,

alpha-cypermethrin,

ivermectin, fipronil

1. Rm_BN_450 D19-01450 Benin LPT Amitraz,

chlorphenvinpos,

alpha-cypermethrin,

ivermectin, fipronil

1. Rm_GH_428 D19-01428 Ghana LPT Amitraz,

chlorphenvinpos,

alpha-cypermethrin,

ivermectin, fipronil

1. Rm_GH_429 D19-01429 Ghana LPT Amitraz,

chlorphenvinpos,

alpha-cypermethrin,

ivermectin, fipronil

1. Rm_NG_430 D19-014230 Nigeria LPT Amitraz,

chlorphenvinpos,

alpha-cypermethrin,

ivermectin, fipronil

1. Rm_NG_431 D19-01431 Nigeria LPT Amitraz,

chlorphenvinpos,

alpha-cypermethrin,

ivermectin, fipronil

1. Rm_UGANDA Rm_Uganda Uganda LPT Amitraz,

chlorphenvinpos,

alpha-cypermethrin,

ivermectin, fipronil

1. Rm_UG_447 D19-01447 Uganda LPT Amitraz,

chlorphenvinpos,

alpha-cypermethrin,

ivermectin, fipronil

1. Rm_UG_448 D19-01448 Uganda LPT Amitraz,

chlorphenvinpos

alpha-cypermethrin,

ivermectin, fipronil

1. Rm_UG_230 D20-02230 Uganda LPT Amitraz,

chlorphenvinpos,

alpha-cypermethrin,

ivermectin, fipronil

1. Rm_UG_250 D20-02250 Uganda LPT Amitraz,

chlorphenvinpos,

alpha-cypermethrin,

ivermectin, fipronil

1. Rm_MX_ElZamora Rm_ElZamora Mexico LIT Amitraz,

chlorphenvinpos,

alpha-cypermethrin,

ivermectin

LPT: larval packet test; LIT: larval immersion test.
