# Supplementary material for: Genomic assessment of targets implicated in Rhipicephalus microplus acaricide resistance
Source: PLoS One. 2024 Dec 5;19(12):e0312074. doi: 10.1371/journal.pone.0312074 (PMC11620669; doi:10.1371/journal.pone.0312074)
Supplement: S2 Table — (DOCX) [file pone.0312074.s020.docx]

Table S2. List of multiplex PCR oligonucleotides used to amplify *R. microplus* targets.

| **Multiplex** | **Target** | **F-oligo name** | **F-oligo sequence** | **R-oligo name** | **R-oligo sequence** | **Amplicon Size** | **Final [primer]** |
| --- | --- | --- | --- | --- | --- | --- | --- |
| 1 | AChE3 | Rm_2_E3-1F | ACGCAAGATACTTACTGGCCTACCAC | Rm_2_E3-1R | CAAGACGAATCAGTCAATTGCTGTC | 267 bp | 0.2 µM |
|  | αAOR | Rm_4_E1-1F | GCACTTGATATGGCATGACCCATATC | Rm_4_E1-1R | ACATACGTGTTTGCTTGAACTTCCTTACG | 1490 bp | 0.4 µM |
|  | βAOR | Rm_5_E1-1F | CAGCTCCACAGGATGAGGCTTG | Rm_5_E1-1R | GGTGAAGTGCGAAGAAGCAGTG | 1584 bp | 0.2 µM |
|  | OCT/TYR | Rm_6_E1-1F | TCTACGATTAGGCCGAGTTCGTCT | Rm_6_E1-1R | TGACTTCACAGACAGACTTTAACAGACACC | 748 bp | 0.2 µM |
|  |  | Rm_6_E2-1F | CACAAGAGATTGGCACATTTACTCGATG | Rm_6_E2-1R | CACGAGCACCACAATGCTTCCTA | 923 bp | 0.2 µM |
| 2 | CYP4W1 | Rm_3_E1-1F | ATGACAATTGTGATGCAACGTAGTGC | Rm_3_E1-1R | AAGCAAATGGCGGGAAGTAGAG | 403 bp | 0.2 µM |
|  |  | Rm_3_E2-1F | TACGTCATTTTGAGCCGAGATTTAATC | Rm_3_E2-2R | CCGAAAGTTCAACACCTTGATCTTTAC | 393 bp | 0.2 µM |
|  |  | Rm_3_E3-4-1F | GGCGTCGTAGATAACTCAAGAATGTC | Rm_3_E3-4-1R | CATCAATCTTATGAAATTGCTGATGCAG | 2321 bp | 0.4 µM |
|  |  | Rm_3_E5-1F | GATTATATCTGAATCTATGCTCTGTTGTCCTTC | Rm_3_E5-1R | CAGGGAATATAAATATACGCTGAGTAATCATTC | 326 bp | 0.2 µM |
|  |  | Rm_3_E6-1F | GCACATTGTGCTCTCTTTGTACCACT | Rm_3_E6-1R | GAATTTTGGGCATATGGCGTG | 613 bp | 0.2 µM |
|  |  | Rm_3_E7-1F | ATGACAGTGATTCGTTGTCGATGC | Rm_3_E7-1R | GAGAGTTCGCACGACTCATCTCACA | 371 bp | 0.2 µM |
|  |  | Rm_3_E8-1F | GCTTCAACAAGTTTGCCTATTAATACGTG | Rm_3_E8-1R | CCACTGTTGCTGGCGTATGTTC | 462 bp | 0.2 µM |
| 3 | AChE2 | Rm_1_E4-1F | GGACTCGCCTAGTAGGTATATTCCATACC | Rm_1_E4-2R | ATTGCACAAAATATTCTACACACGCAC | 259 bp | 0.2 µM |
|  | Voltage-gated sodium channel | Rm_7_E3-5-1F | GTTTATACGGCTAATTAAATAAGGTGCTCATATC | Rm_7_E3-5-1R | TCTTGGCGATCTCGGTAGTTCATC | 1279 bp | 0.2 µM |
|  |  | Rm_7_E6-1F | GAGTTGAAGCAGTCTCTTGTGCGC | Rm_7_E6-1R | CTGTTCGAGTTAGCGTGCAGCTG | 395 bp | 0.2 µM |
|  |  | Rm_7_E7-8-1F | CTTGCTCCATCATGCGGACATT | Rm_7_E7-8-1R | CGACCATACCAGACGTTGCAAC | 2269 bp | 0.2 µM |
|  |  | Rm_7_E9-10-1F | CTCACTGGCGGCCATACTTAAAC | Rm_7_E9-10-1R | CTAACGAAGATACAGCGTTTAGAAATCTGAG | 2595 bp | 0.2 µM |
| 4 | AChE2 | Rm_1_E1-1F | ATACACGTGATATCACCTCGTCCAGC | Rm_1_E1-1R | AACGTCACGAAATAAGAAAGAGCCG | 695 bp | 0.4 µM |
|  | AChE2 | Rm_1_E3-1F | CTCGCTGGCAGTATATTATGTGAGAAAAG | Rm_1_E3-1R | GGATGTACAGCACCATGAAATGTCG | 1243 bp | 0.2 µM |
|  | Voltage-gated sodium channel | Rm_7_E16-17-1F | CTCTCGTGTTTCACGAGTATCCTTTG | Rm_7_E16-17-1R | CAGGCCTTGGGAGATGAGTGAG | 3118 bp | 0.4 µM |
|  |  | Rm_7_E18-19-1F | CTTTCATCTCTGCCGTGGTCTGTAG | Rm_7_E18-19-1R | CCTGAACCCTGCTAACAAAATGATTG | 2739 bp | 0.2 µM |
|  |  | Rm_7_E20-21-1F | GGTTACATCATATCTCATTGCCACGC | Rm_7_E20-21-1R | CAACGTCAACCACAACAACTCTGATG | 1653 bp | 0.2 µM |
|  |  | RM_7_E24-1F | TGTATGCACACAAGGACCACGA | Rm_7_E22-24-1R | CACTCGCACACTGGTAGGCAGTC | 254 bp | 0.2 µM |
|  |  | Rm_7_E1-2-1F | AACGGCGTCTGTAAATGCGAAC | Rm_7_E1-2-2R | CTCTTCCATGTTCTGCGACTCAAC | 2199 bp | 0.2 µM |
| 5 | Carboxylesterase | Rm_CBE_E1-1F | GTTCAGCGATTTGTTTCACCCTC | Rm_CBE_E1_1R | CCGAGCACTGTAATGACTGTTC | 414 bp | 0.2 µM |
|  |  | Rm_CBE_E2-1F | CAGCTAATTTCAGAAACGGCTCACTAC | Rm_CBE_E2-1R | AATGAAATGCGAGTGAACTTGTTCATG | 1500 bp | 0.4 µM |
|  |  | Rm_CBE_E3-1F | CTTTATTGATTGCTCTTGCGTCACG | Rm_CBE_E3-1R | CGAATGTGCGAAACTGTATATACGTATAAC | 284 bp | 0.4 µM |
|  | AChE3 | Rm_2_E2-1F | TGGTAATCTAGTGTTTGCTTGGGTAGACAG | Rm_2_E2-1R | CTGTACTTAGCAAGCTGCAAATCTTTACG | 1882 bp | 0.2 µM |
| 6 | CYP41 | Rm_CYP41_E1-1F | AGCTTGATAGTGTAAAATATTTTGATCCGTG | Rm_CYP41_E1-1R | AAACGCTCTGTGATTCCCATTCTTC | 250 bp | 0.2 µM |
|  |  | Rm_CYP41_E5-1F | GTACATAAAGCACTGCTTGCTATATATCTGTC | Rm_CYP41_E5-1R | CTCCGTGGATATGCTGTGAATTATTTC | 371 bp | 0.2 µM |
|  |  | Rm_CYP41_E6-8-1F | TTGCTGTCCCAGTAGTTTTCGTTG | Rm_CYP41_E6-8-1R | GTGTTCTGTGTTCGGTAATACGTGG | 1625 bp | 0.2 µM |
|  |  | Rm_CYP41_E9-10-1F | TAAGCCGAAAGTGACATAGTTGAAGC | Rm_CYP41_E9-10-1R | GTTTCAGCAAAGAAACGAGCCTCTA | 2069 bp | 0.2 µM |
|  | AChE2 | Rm_1_E2-1F | TAGTTTACTACGGGATGAAGGAAAAGTGG | Rm_1_E2-1R | ACTGCCTACAACATCCTTATCTTAATCCTAAT | 293 bp | 0.2 µM |
| 7 | CYP41 | Rm_CYP41_E2-4-1F | CACACTAGATTTAAGCGAAACTGAGTTTC | Rm_CYP41_E2-4-1R | CCGCTTAGCTGTTATTTACTAATGAAAAATTGTT | 535 bp | 0.2 µM |
|  |  | Rm_CYP41_E11-1F | TACCTTGCAGCCTTCCAATTCTTTG | Rm_CYP41_E11-1R | ATTCTACGACGCGTATTCTCTAACTCTG | 372 bp | 0.2 µM |
|  |  | Rm_CYP41_E12-14-1F | CATTCATCGGTTCACATGCTCG | Rm_CYP41_E12-14-1R | ACAAGTATGTGGTGCCTAATTAGAGTTG | 2405 bp | 0.2 µM |
| 8 | GABA-gated chloride channel | Rm_GABA_E2-1F | GGGTCTTGTATCTGTATGCATTCTTTAATG | Rm_GABA_E2-1R | CAATAGATGAAGATTCACGAAAGCCA | 392 bp | 0.2 µM |
|  |  | Rm_GABA_E3-1F | GAGTCGCGCGACTTAAAACG | Rm_GABA_E3-1R | CTTCATGACGAATACTCGCCAAAGAG | 687 bp | 0.2 µM |
|  |  | Rm_GABA_E4-5-1F | GAAAATTCGAAGAGAGGCTCTGATGC | Rm_GABA_E4-5-1R | CGAAACATTAAAACAGATAATAGCAGGTAGTGG | 1724 bp | 0.2 µM |
|  |  | Rm_GABA_E6-1F | GCAGCTCACTAATACTCTTACTCTTGCTC | Rm_GABA_E6-1R | ACGAACGACACACCTGTTACG | 897 bp | 0.2 µM |
| 9 | GABA-gated chloride channel | Rm_GABA_E1-1F | CTCGAAGAATCCGAGTACTCACT | Rm_GABA_E1-1R | TCGTCTGTCACAAGCAGTCATG | 619 bp | 0.1 µM |
|  |  | Rm_GABA_REF_E2-1F | ATCGCAACTGTGCTTCCTTATC | Rm_GABA_REF_E2-1R | GGTCTGACATTAACACATATTCAAC | 357 bp | 0.2 µM |
|  |  | Rm_GABA_REF_E4-5-1F | GTGACATCTCCTTTACATTGCTG | Rm_GABA_REF_E4-5-1R | CTAACTGATGCCTGTATGTGGC | 511 bp | 0.1 µM |
|  | Voltage-gated sodium channel | Rm_7_E22-24-2F | CGCTTTCTGCTGTCTTATGTATGCC | RM_7_E22-23_1R | CTATGTGTATCACGGTCTCGGTA | 1841 bp | 0.2 µM |
| 10 | Voltage-gated sodium channel | Rm_7_E11-12-4F | GTAACTTAGTATGAAAAACACATGGTTACG | Rm_7_E11-12-5R | CAGGCGTGTGGAGGAACGATACAATCGAATG | 2500 bp | 0.2 µM |
|  |  | Rm_7_E13-1F | CTTACATTGCATTTTGAATCTGCGTG | Rm_7_E13-1R | TGAGTACAGAATAATAATGTGCTGGCAATG | 448 bp | 0.2 µM |
|  |  | Rm_7_E14-15-4F | GTCTTGCTAATATTTCTACTTCCCTTCAC | Rm_7_E14-15-4R | CTTGACTACCGGCCAGAATATCCCTTGTG | 1713 bp | 0.2 µM |
|  | GABA-gated chloride channel | Rm_GABA_REF_E3-1F | CAGCTGTSTAATGACGTTATATCC | Rm_GABA_REF_E3-1R | TTTTAATGAGCGTTCGAATTTG | 314 bp | 0.2 µM |
| 11 | CYP4W1 | Rm_3_E1-1F | ATGACAATTGTGATGCAACGTAGTGC | Rm_3_E3_1-1R | AAGCGTTATGAAACAGATTGACATG | 1115 bp | 0.2 µM |
|  |  | Rm_3_E5_1-1F | GAACACGTGTAAGTGGACCTATG | Rm_3_E5_1-1R | GACATTCTTGAGTTATCTACGACGCC | 292 bp | 0.2 µM |
|  | Glutamate-gated chloride channel | Rm_8_E1-1F | CTCATCACAGGAATGAGCGAACAC | Rm_8_E1-1R | GCTGCTAGACCATCACATTTAGTC | 502 bp | 0.2 µM |
|  |  | Rm_8_E4-1F | GCTTAGGTTACGCTAAAGGTCAC | Rm_8_E4-1R | CACTGCCAAGATGAGCTGTC | 467 bp | 0.2 µM |
|  |  | Rm_8_E6-E7-1F | CACAACAAGTATGACACGTATATGAAC | Rm_8_E6-E7-1R | AATGAAGAGCTGGTTGAATTGAGAC | 2175 bp | 0.2 µM |
| 12 | Voltage-gated sodium channel | Rm_7_EM_1-2-5F | GAACGTATTGCACGCTTTGTAGAATA | Rm_7_EM_1-2-4R | CTTTGTGAGCTTGCACGGTTTGTATATACGC | 1550 bp | 0.2 µM |
|  | Glutamate-gated chloride channel | Rm_8_E2-1F | AGAATACGAGATGGCCAAAATTAC | Rm_8_E2-1R | GGCAAAATGTCACAAGCAATC | 475 bp | 0.2 µM |
|  |  | Rm_8_E3-1F | GACACATCGCTAGGTAGGGC | Rm_8_E3-1R | GCTAGTATTTGCGCGGTAATG | 540 bp | 0.2 µM |
|  |  | Rm_8_E5-1F | CGTGAAGCTATCCAGTATTATCCTC | Rm_8_E5-1R | CTAACCTACTTCGGCGGATCA | 349 bp | 0.2 µM |
|  |  | Rm_8_E8-1F | GTGCACTGTTCTGTATTCTCGAC | Rm_8_E8-1R | TTGTCGGAGCCTCTTACAGCT | 621 bp | 0.2 µM |
| 13 | αAOR | Rm_4_E3-1F | CTACTATATGTTACCATGCGAACAATC | Rm_4_E3-1R | GCCGTGTATCCACGAAGCGATG | 300 bp | 0.2 µM |
|  | Voltage-gated sodium channel | Rm_7_E4m_1F | AAAGTAATCGTGAAACGCTTCTC | Rm_7_E6m_1R | CTGTTCGAGTTAGCGTGCAGCTG | 2500 bp | 0.2 µM |
|  |  | Rm_7_10m-2F | CATCTAAACGAAGTTAATGCTATTG | Rm_7_10m-2R | CTGGTATTAGCACTCCATACAG | 480 bp | 0.2 µM |
|  |  | RM_7_E20-1F | CATGCCCTTATAATCTCATTACCATTG | RM_7_E20-1R | AATGGTTCGAGTGCCACATGTG | 600 bp | 0.2 µM |
|  |  | RM_7_E21-2F | CTACAATAATCCCAACTCGCG | RM_7_E21-2R | TTATCTATGACACACTGCCAACCTC | 620 bp | 0.2 µM |

AChE: Acetylcholine esterase; αAOR: α-adrenergic-like octopamine receptor; βAOR: β-adrenergic-like octopamine receptor; OCT/TYR: octopamine–tyramine receptor; GABA: Gamma-aminobutyric acid.
