# Supplementary material for: Genomic assessment of targets implicated in Rhipicephalus microplus acaricide resistance
Source: PLoS One. 2024 Dec 5;19(12):e0312074. doi: 10.1371/journal.pone.0312074 (PMC11620669; doi:10.1371/journal.pone.0312074)
Supplement: S3 Table — (DOCX) [file pone.0312074.s021.docx]

**Table S3**. Amino acid substitutions in acaricide target genes in *R. microplus* isolates from this study.

**Acaricide Target Nucleotide Amino Acid (AA) Number**

**Class** **substitution** **substitution of isolates**

**(acaricide)** **with the AA**

**substitution**

Formadines βAOR gene C311T A104V 1

(amitraz) T955G C319G 5

T955K G/C 2

αAOR gene C83S S/C (S28C) 6

A494R N/S (N165S) 6 G511R R/G (G171R) 13

G1359N E/D (E453D) 27

OCT/TYR gene A22C T8P* 1

A22M T/P 18

A43G I15V* 3

A43R I/V 30

A58G T20A* 6

A58R T/A 27

T65C L22S* 1

T65Y S/L 13

T77C M26T 12

T77Y T/M 22

G881A G294D 1

G881R D/G 13

G931C E311Q 1

G931S Q/E 23

A1240C N414H 1

A1240M N/H 25

Phenyl- GABA-Cl gene G52R I/V (V18I) 15

pyrazoles

(Fipronil)

Glu-Cl gene - - -

Macrocyclic Glu-Cl gene - - -

lactones

(doramectin; GABA-Cl gene - - -

ivermectin)

Organo- AChE gene (AChE2) T35C V12A 4

phosphates T35Y A/V 5

(chlorfen- C52M I/L (L18I) 4

vinphos) A64G K22E 3

A64R K/E 5

G76R T/A (A26T) 4 C80G T27R 1

C80S T/R 1

C80R K/R (T27K/R) 6

A128R H/R (H43R) 5

G148T A50S 3

G148K A/S 5

G157R I/V (V53I) 4

G171T E57D 6

G171K E/D 3

T247K A/S (S83A) 3

C251Y A/V (A84V) 5

C584Y; C585Y P/L (P195L) 6

A739R T/A (T247A) 9

A749W K/M (K250M) 5

A749R K/R (K250R) 6

C806A; M807C T269N 3

C806M N/T 3

R827G Q/R276R 21

R868G T/A290A 11

R886G N/D296D 17

R886A; T888C N/D296N 1

G889A V297I* 37

M896C D/A299A 19

G908C R303T 10

G908S T/R 18

G949T; V317F 1

G951K

G949K; G951T V/F 1

A1063W; N355Y 3

T1065Y

A1063T; N/Y 20

T1065C

T1090A S364T* 37

T1094W Y/F (F365Y) 6

T1186Y P/S (S396P) 9

C1234T H412Y* 37

A1319R K/R (K440R) 6

A1359C E453D 3

A1359M E/D 19

G1403A R468K* 37

A1467T E489D 3

A1467W E/D 19

T1476C G492S 3

T1476Y S/G 16

G1505A R502K 3

G1505R K/R 20

C1560A F520L 1

C1560M L/F 22

AChE gene (AChE3) A162G I54V * 36

G257A R86Q* 37

G409A V137I* 23

G409R I/V 14

C1476G I492M* 37

A1642G T548A* 23

A1642R T/A 14

Synthetic VGSC gene C1471N R/S (S482R) 28

Pyrethroids G2019A G655S 3

(deltamethrin; G2019R S/G 15

alpha- G2575N K/N (K837N) 14

cypermethrin) C2837A L925I * 23

C2837M I/L 3

C3700T A1186V 29 T3699Y A/V 4

CBE gene R25A; Y27T I/V9I 23

R25G; Y27C I/V9V 2

R91A T/A31T 24

A91G; C93T T/A31A 2

C/G175C/G† P59 28

C175S P/A 8

C175G P59A 1

S181C L/V61L 23

S181G L/V61V 2

S222G S/R74R 23

S222C S/R74S 2

M227A Q/P76Q 22

M227C Q/P76P 2

Y302T A/V101V 18

Y302C A/V101A 9

R334A I/V112I 18

R334G I/V112V 9

S360C N/K120N 18

S360G N/K120K 8

R469G I/V157V 20

R469A I/V157I 2

R821G N/S274S 18

R821A N/S274N 9

R/N870R/N† K290 30

R/N870N K/N 7

G1000R T/A (A334T) 11

A1048G; M350T 17

T10049C

Y1070T P/L357L 17

Y1070C P/L357P 7

G1120R N/D (D374N) * 14

R1132A T/A378T 17

R1132G T/A378A 8

A1201C; R401Q 17

G1202A

R/N1302R/N† R434 29

R/N1302N R/S 8

K1395G Q/H465Q 17

K1395T Q/H465H 8

Y1436T P/L479L 17

Y1436C P/L479P 8

A1555G I519V 2

Cytochrome G220A A74T* 37

P450 A1188M E/D (E396D) 4

(CYP4W1) T1616A I539K* 37

T1631A V544E* 37

Cytochrome A464R H/R (H155R) 7

P450 A671G T224A* 37

(CYP41) A731T T/S (T244A)* 1

A743R N/D (N248D)* 10

T793N E/D (D266E) 35

A866T T289S 1 A866W T/S 6

G911R I/V (V304I) 2

G1164C G388A* 37

A1166T T389S* 37

T1451C C484R* 37

AChE: Acetylcholine esterase; βAOR: β-adrenergic-like octopamine receptor; αAOR: α-adrenergic-like octopamine receptor; OCT/TYR: octopamine–tyramine receptor; *Previously reported amino acid substitutions; GABA: Gamma-aminobutyric acid; †No distinct wild-type genotype.
